# Detection and Architecture of Small Heat Shock Protein Monomers

**DOI:** 10.1371/journal.pone.0009990

**Published:** 2010-04-07

**Authors:** Pierre Poulain, Jean-Christophe Gelly, Delphine Flatters

**Affiliations:** DSIMB, Inserm UMR-S 665 and Université Paris Diderot - Paris 7, INTS, Paris, France; Institute of Infectious Disease and Molecular Medicine, South Africa

## Abstract

**Background:**

Small Heat Shock Proteins (sHSPs) are chaperone-like proteins involved in the prevention of the irreversible aggregation of misfolded proteins. Although many studies have already been conducted on sHSPs, the molecular mechanisms and structural properties of these proteins remain unclear. Here, we propose a better understanding of the architecture, organization and properties of the sHSP family through structural and functional annotations. We focused on the Alpha Crystallin Domain (ACD), a 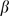 sandwich fold that is the hallmark of the sHSP family.

**Methodology/Principal Findings:**

We developed a new approach for detecting sHSPs and delineating ACDs based on an iterative Hidden Markov Model algorithm using a multiple alignment profile generated from structural data on ACD. Using this procedure on the UniProt databank, we found 4478 sequences identified as sHSPs, showing a very good coverage with the corresponding PROSITE and Pfam profiles. ACD was then delimited and structurally annotated. We showed that taxonomic-based groups of sHSPs (animals, plants, bacteria) have unique features regarding the length of their ACD and, more specifically, the length of a large loop within ACD. We detailed highly conserved residues and patterns specific to the whole family or to some groups of sHSPs. For 96% of studied sHSPs, we identified in the C-terminal region a conserved I/V/L-X-I/V/L motif that acts as an anchor in the oligomerization process. The fragment defined from the end of ACD to the end of this motif has a mean length of 14 residues and was named the C-terminal Anchoring Module (CAM).

**Conclusions/Significance:**

This work annotates structural components of ACD and quantifies properties of several thousand sHSPs. It gives a more accurate overview of the architecture of sHSP monomers.

## Introduction

Small Heat Shock Proteins (sHSPs) belong to the large superfamily of protein chaperones. More precisely, they present an ATP-independent chaperone-like activity since they bind (but not refold) non-native proteins [1]–[3]. Under stress conditions (high temperature, oxidative stress, *etc.*), they thereby prevent the irreversible aggregation of misfolded proteins. Their key roles in the cell have been demonstrated through the description of numerous mutations in human sHSPs found to be involved in severe pathologies (desmin-related myopathy [4], neurodegenerative diseases, distal hereditary motor neuropathy, cataract or tumors) [5]–[7]. sHSPs are ubiquitous proteins found from few in archaea, bacteria or yeast [8], to a dozen in humans [9]–[11] and more than 15 in plants [12]. In higher multicellular eukaryotes, each sHSP has a specific subcellular localization and/or tissue distribution [13].

An sHSP monomer has a molecular weight between 15 and 40 kDa and shares a conserved domain of 80 to 100 amino acids called the Alpha Crystallin Domain (ACD) [14], [15]. Most sHSPs are structurally organized as large oligomers. Upon cell stress, they adjust their oligomeric state to bind misfolded substrates [3], [5], [16]–[8]. Despite their critical role in the cell, the mechanisms of sHSP function are not well known and obtaining experimentally resolved structures for this family is still challenging [17], [19]. This difficulty is due to the high plasticity of sHSP quaternary structures since these proteins are able to change their oligomeric state under different conditions, to exchange their subunits or to show polydisperse properties [3], [20].

To date, only six sHSP structures have been resolved at atomic resolution: namely HSP 16.5 (PDB 1SHS) [16], HSP 16.9 (PDB 1GME) [17], TSP 36 (PDB 2BOL) [18], HSP A (PDB 3GLA) [21], HSP 20 (PDB 2WJ5) [22] and 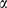
B crystallin (PDB 2WJ7) [22]. These homopolymeric structures display a large variation in oligomer states (size and shape), but their monomers all share a common 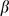 sandwich fold composed of two sheets with respectively four (termed 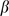
2, 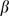
3, 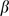
8, 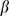
9) and three (termed 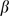
4, 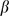
5, 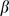
7) 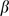 strands. The second 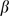 sheet is characterized by a large L57 loop, linking strands 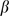
5 and 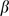
7, and involved in dimerization in HSP 16.5 [16], HSP 16.9 [17], TSP 36 [18] and HSP A [21]. The 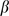 sandwich region is flanked by the N-terminal and C-terminal regions, described as variable in both length and amino acid composition [8], [14]. These structural data have highlighted that the well-conserved 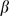 sandwich fold is in fact associated with the conserved sequence region known as ACD.

*In vitro* studies showed that ACD is essential for the construction of dimers and higher-order assemblies, but also for the function of sHSPs [23]–[25]. The N-terminal region, described as disordered, is associated with poorly conserved sequences and is generally hydrophobic [20]. It is involved in substrate binding and in higher oligomeric assembly [19], [26]. The C-terminal region, flexible and unstructured, shows sequence variability with a polar tendency. This region participates in stabilizing and solubilizing the oligomeric assemblies [27]. A well-conserved motif in the C-terminal region has been shown to be involved in the inter-dimer interactions of HSP 16.5 [16] and HSP 16.9 [17]. The most divergent fragments in the sequence (the L57 loop in ACD, the N-terminal and C-terminal regions) are thought to be responsible for the large variability observed in oligomeric structures. For instance, the insertion of a peptide in the HSP 16.5 N-terminal sequence leads to either larger symmetric or polydisperse assemblies [28].

In contrast to the few resolved structures, numerous sHSP sequences are available in the UniProt databank [29] and have been annotated as belonging to the small Heat Shock Protein (or HSP20) family, primarily based on sequence information. sHSPs are associated with the PROSITE [30] profile PS01031 [15]. This profile is based on a sequence alignment of a conserved domain of about 100 residues related to ACD [14], [31]. Proteins identified as sHSPs are also linked to the Pfam [32] motif PF00011, which is built from an alignment of a region of 115 amino acids roughly corresponding to ACD and the C-terminal region.

In early studies of sHSPs, sequence information was extensively used to explore evolutionary analyses [14], [15], [33], [34], to discover new human sHSPs [10] or, more generally, to link sequence, structure and/or function in the sHSP family [3], [15], [35]. For instance, on the basis of an alignment of 344 unique sequences, Fu and Chang identified a highly conserved P-G doublet in non-animal sHSPs [36]. In another work based on the alignment of 26 sHSP sequences, the impact of substituting non-conserved residues in the 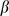
3 strand on the assembly or functional properties of 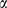
B crystallin was assessed [37].

These previous works were all based on limited sets of sequences (less than 350 proteins) compared by multiple sequence alignments. However, this approach is not suitable to study several thousand sHSPs since these proteins are heterogeneous in their sequence, albeit the conservation of ACD [12], [15], [33], [34]. For this reason, no systematic delineation and analysis of the sHSP monomer with respect to its structural topology has been yet established.

Here, we used an original approach that consists in building a Hidden Markov Model (HMM) profile based on available structures and structurally well-annotated sequences and then in enriching it using an iterative procedure. This method leads to a robust delineation and an accurate identification of ACD in sequences of the UniProt databank. We defined three regions from a structural point of view. ACD is the region delimited from the 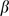
2 strand to the 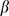
9 strand. The N-terminal is the region preceding the 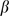
2 strand and the C-terminal is the region following the 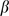
9 strand. We identified a specific fragment in the C-terminal region that we named the C-terminal Anchoring Module (CAM). This fragment starts after ACD and includes a conserved motif. Residues that follow were defined as the C-terminal tail. Finally, we indicated specific sequence properties for sHSPs belonging to taxonomic groups such as plants and animals or previously studied groups such as class A bacteria and class B bacteria [36], [38]. With this structure-based procedure, we analyzed an exhaustive set of sHSP sequences, characterized their architectural features and established sequence/structure relationships.

## Results

**Figure 1:**
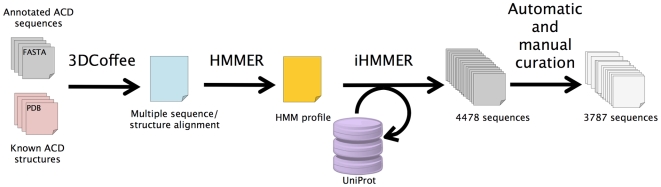
Flowchart of the pipeline set up in this work.

The global procedure defined in this study is plotted in Fig. 1 and was based on three steps: (1) the constitution of an initial multiple structure/sequence alignment of known ACD structures and well-annotated sequences, (2) the construction of a Hidden Markov Model (HMM) profile of ACD based on the previous alignment and enriched with an iterative procedure using the HMMER software, and finally, (3) an exhaustive survey of the UniProt databank to detect ACDs.

### Multiple structure/sequence alignment

**Figure 2:**
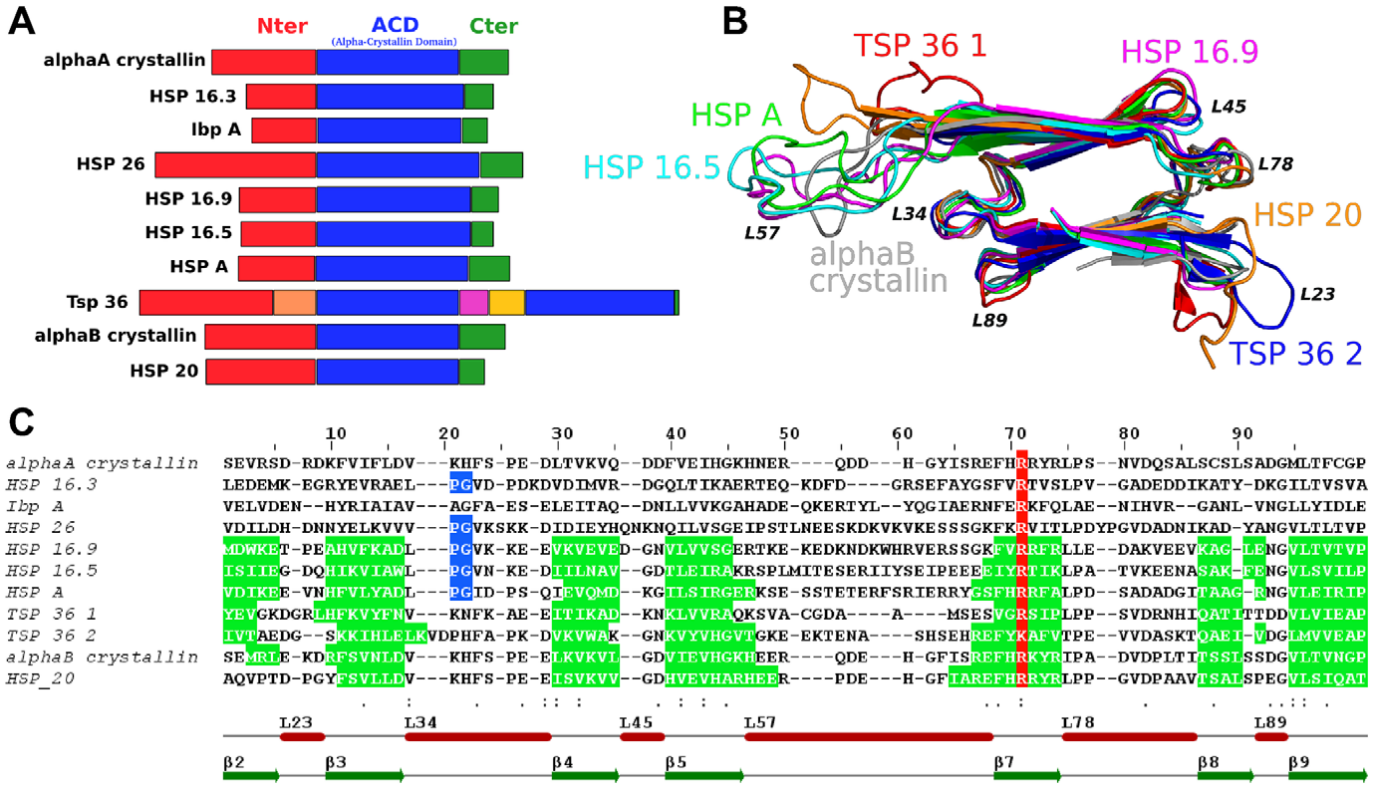
Reference sHSP sequences, superposition of ACD structures and initial multiple structure/sequence alignment. (A) Scheme of the 10 sequences used for the construction of the profile with delineation of the three regions: the N-terminal region in red, ACD in blue and the C-terminal region in green. For the TSP 36 sequence that contains two ACDs, linkers [18] are shown in orange, pink and yellow (B) Structural alignment of known ACDs with in-between-strand loop annotation, 3D representation made using PyMOL [59] (C) Multiple structure/sequence alignment from 3DCoffee on the ACD region. The secondary structure assignment is shown in green, 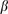 strands are annotated as green arrows and loops as red bars. ACD is subdivided into three parts: the 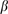
2–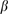
5 zone, the L57 loop and 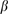
7–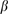
9 zone. The conserved arginine [15], [40] is shown in red and the P-G doublet [15], [36] in blue. Alignment representation performed using Jalview [60].

In step (1), the 11 ACDs used as references are representative of the sHSP family (animals, plant, archae, bacteria, fungi). The nomenclature of the 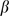 strands (from 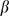
1 to 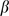
10) in the 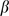 sandwich fold was defined relative to the first sHSP structure to be elucidated, HSP 16.5 [16]. Here, we defined ACDs from the beginning of the 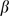
2 strand to the end of the 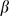
9 strand. The 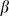
2 strand is the first structural element shared by all available X-ray structures, except for mammalians HSP 20 and 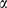
B crystallin (in only one monomer of the dimeric structure). It is also involved in the dimerized structure of HSP 16.5, HSP 16.9 and HSP A. The 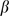
9 strand is the last strand of ACD and is found in all known ACD structures. The 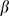
1 strand, not assigned in other structures, is localized in the N-terminal region for our definition of ACD. Strands 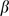
6 (in the L57 loop) and 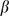
10 (corresponding to the conserved motif in the C-terminal region) were only assigned as 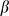 strands in the context of subunit interactions (i.e. for HSP 16.5 and HSP 16.9 structures). Fig. 2A shows a schematic diagram of the reference sequences. As reported in the literature, the length of ACD is more constant than the other two regions. Superposition of the ACD structures of the common 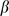 sandwich fold also showed that ACD length is relatively well conserved (Fig. 2B). A pairwise structural alignment of ACD resulted in a low root mean square deviation (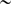
1.5 Å) demonstrating a strong structural signature in this region. In contrast, the corresponding ACD sequences show only moderate identity (25–30%). Fig. 2C presents the multiple structure/sequence alignment obtained with the 3DCoffee web server [39]. For the seven ACD structures, the 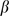 strand assignments were well superimposed. Conserved motifs or residues are also represented, such as the P-G doublet for non-animal sHSP [15], [36] and the arginine associated with human pathologies in the 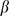
7 strand [15], [40]. From this alignment, we subdivided ACD into three zones (the 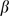
2–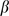
5 zone, the L57 loop and the 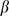
7–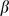
9 zone) to analyze separately the most variable part in ACD (*i.e.* the L57 loop).

### Iterative HMM profile construction

Based on this initial structural alignment, we built in step (2) a HMM profile iteratively enriched with other sequences retrieved from the UniProt databank and identified with high confidence as sHSPs (see Materials and Methods). By doing so, we were able to detect and delineate ACD more accurately, since HMM derived from the structural alignment profile performed significantly better than HMM derived only from sequence alignments [41], [42]. At the end of this procedure, the generated HMM profile was named ACDP09.

### ACD detection

Finally, in step (3), 4478 UniProt sequences showed a positive match using the ACDP09 profile (with E-value 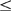 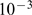).

### sHSP identification and region delineation

We assessed the quality of our results by comparing the retrieved sequences and the proteins annotated as sHSP in UniProt. Results are shown in Table 1. Four annotations were tested, namely family:“small heat shock protein”, family: “HSP20”, “prosite PS01031”, and “pfam PF00011”. The annotations “small heat shock protein” and “HSP20” are common names for sHSPs and are in fact synonymous because they were associated with the same sequences and 95% of these sequences were included in our dataset. The “prosite PS01031” and “pfam PF00011” annotations were assigned to more sequences and 92% and 91%, respectively, of them were also found in our dataset.

**Table 1:** Overlap between sHSP annotated sequences in UniProt and sequences detected with ACDP09.

annotation	number of UniProt sequences	% of sequences in common with ACDP09 results
family:“small heat shock protein”	4140	95
family:“HSP 20”	4140	95
“prosite PS01031”	4499	92
“pfam PF00011”	4377	91

Sequences not retrieved by our method but labeled as sHSP with one of the four annotations above were too small (50 residues or less) or defined as fragment (with a potentially incomplete ACD pattern). Sequences identified by us as sHSP but not such annotated are sequences recently integrated in UniProt and not yet curated (either manually or automatically). Two months after this initial annotation comparison, about 350 more sequences were indeed labeled as sHSP in UniProt. In conclusion, the ACDP09 profile is in good agreement with known profiles integrated in PROSITE or Pfam databanks. It is more specific than the latter two, since incomplete ACD were not allowed in our methodology. The sensitivity is at least equivalent to the other profiles because clearly identified and annotated sHSPs were detected with ACDP09. Furthermore, the accurate ACD detection induced the delineation of the different regions of sHSP sequences and the different zones within ACD.

Finally, from the initial 4478 retrieved sequences, we selected complete proteins with only one ACD and a high level of protein existence evidence (see Materials and Methods section). This final dataset (named sHSPdata09 and provided as Dataset S1) included 3787 sequences of which 549 (15%) were reported in animals, 688 (18%) in plants, 612 (16%) in class A bacteria (bacA), 206 (6%) in class B bacteria (bacB), 123 (3%) in fungi, 85 (2%) in other eukaryotes (other), 1378 (36%) in bacteria that were neither class A nor class B bacteria (bacOther) and 146 (4%) in archaea (for group definition, see Materials and Methods). We analyzed in detail the full sHSPdata09 dataset and the four largest and most homogeneous groups, namely animals, plants, bacA and bacB. These four groups represent 55% of sHSPdata09. From the delineation of ACD, we easily determined the flanking N-terminal and C-terminal regions. Our dataset shows that the length of the N-terminal region varied greatly (with an average length of 53
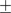
35 residues), even within each group. The N-terminal region is a highly heterogeneous region for which structural information is often missing (except for HSP 16.9 and TSP 36 structures). Thus, it still remains difficult to build a relevant multiple alignment or profile to identify conserved residues or motifs for this region.

### The ACD region

**Figure 3:**
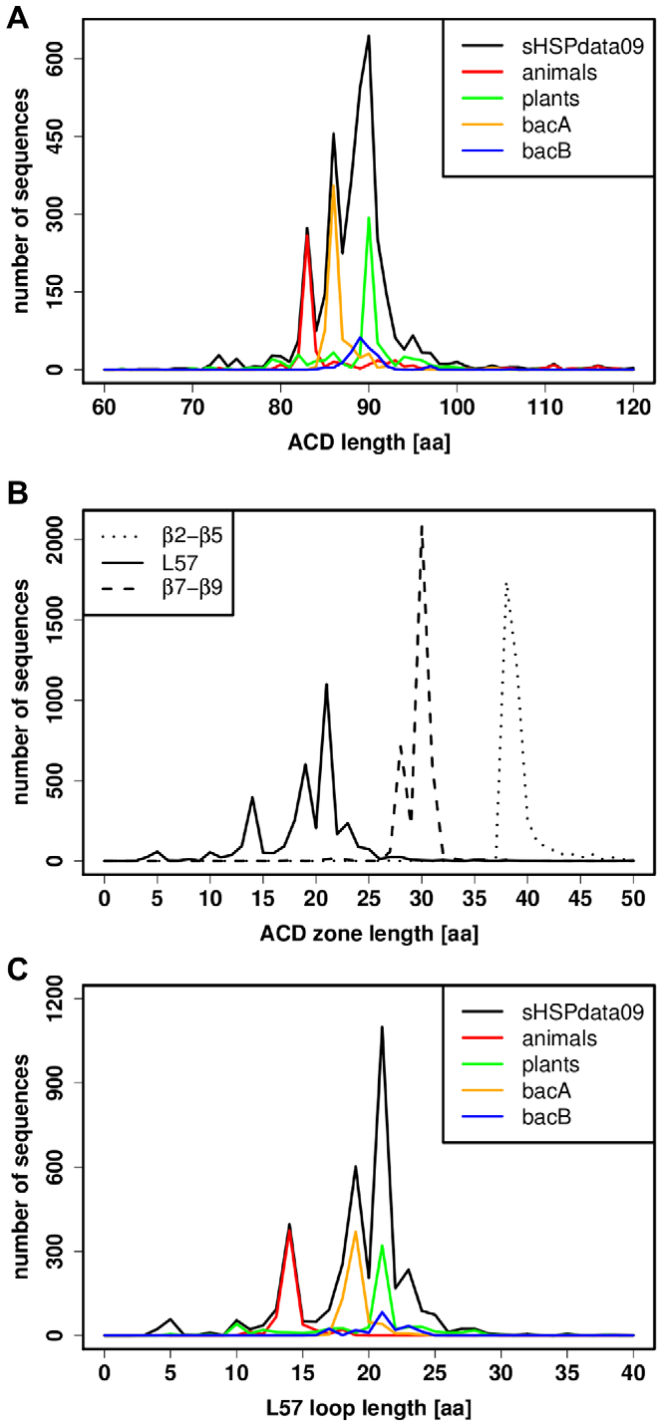
Length distributions of ACD and ACD zones. (A) Length distribution of ACD for sHSPdata09 sequences in black, animals in red, plants in green, bacA in orange and bacB in blue. Sequence lengths correspond to a number of amino acids (aa). (B) Length distribution of zones (inside the ACD) for sHSPdata09 with the 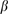
2–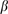
5 zone in dotted line, the L57 loop in solid line and the 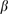
7–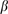
9 zone in dashed line (C) Length distribution of the L57 loop for sHSPdata09 in black, animals in red, plants in green, bacA in orange and bacB in blue.

The ACD region is the structural basis of the sHSP family and is well described in the literature [14], [23]. We found an average ACD length of 90
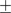
10 residues, a value that corroborates previous studies [5], [31]. The length distribution of ACD is presented in Fig. 3A for sHSPdata09 sequences. It exhibits three peaks in a very narrow range, as 90% of sequences have an ACD length of between 82 and 100 residues. However, very large differences can occur between distributions of specific groups and are shown for animals, plants, bacA and bacB in Fig. 3A. For animals, the ACD length distribution was centered at 83 residues. Particularly, the subset of the 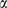 crystallin sHSPs (13% of animals) had an ACD length of exactly 83 residues, although the full sequence length displayed more heterogeneity. In contrast, the ACD length distribution of plants and bacA shows a peak at 90 and 86 residues respectively. For bacB, the distribution shows a broad peak at 89 residues. These results are in line with the differentiation of bacA and bacB [38]. To explore ACD further, we subdivided this region into three zones: the 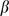
2–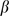
5 zone, the L57 loop and the 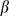
7–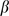
9 zone. The length distribution of the three zones is represented in Fig. 3B. For the 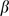
2–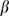
5 zone, the distribution is centered at 38 residues. For the L57 loop, the distribution shows a first peak at 14 residues and a second, larger peak, at 21 residues. Finally, for the 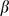
7–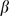
9 zone, the distribution shows two peaks at 28 and 30 residues. The L57 loop was the smallest zone in ACD but it was responsible for most of the variability in ACD length. A closer look at the length distribution of the L57 loop (Fig. 3C) clearly shows that animal sHSPs are generally associated with a very small L57 loop (13 residues on average). Other groups (plants, bacA and bacB) had a larger L57 loop, with a less uniform distribution for bacB and an intermediate L57 loop length for bacA. Among animals, the 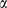 crystallin sHSPs had a 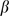
2–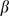
5 zone, L57 loop and 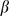
7–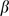
9 zone lengths of 38, 14 and 31 residues, respectively. In addition, hydropathy scores [43] for the three zones clearly confirmed the more hydrophilic nature of the L57 loop compared to the 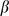
2–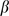
5 and 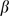
7–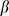
9 zones [44] (data not shown).

**Figure 4:**
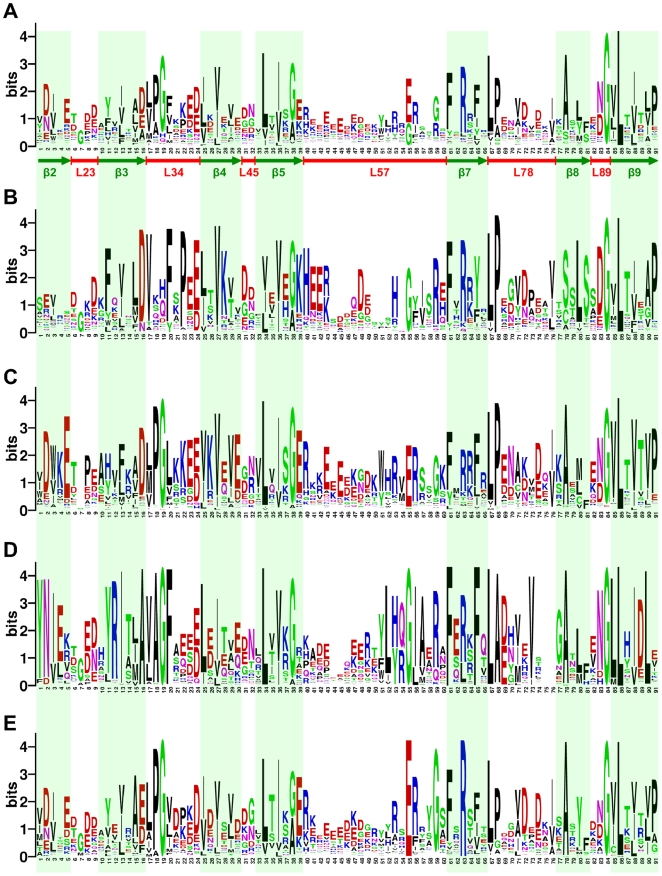
Logo representation of the ACD profile. The height of each letter is proportional to the information content of residues that matched ACDP09 profile positions. Amino acids are colored according to their chemical properties: acidic (D,E) in red, basic (K,R,H) in blue, polar (G,S,T,Y,C) in green and (N,Q) in purple, hydrophobic (A,V,L,I,P,W,F,M) in black. Logo representation for (A) sHSPdata09, (B) animals, (C) plants, (D) bacA and (E) bacB. The 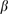 sheets and loops are annotated in green and red, respectively.

The logo representation of ACD in Fig. 4 highlights the most conserved residues that match the positions of the ACDP09 profile. For sHSPdata09 (Fig. 4A), some residues or motifs were highly conserved in the family, for instance, G/A at the end of the 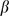
5 strand, F-X-R in the 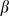
7 strand, L-P/A at the beginning of the L78 loop, N/D-G-hydrophobic-L between the L89 loop and the 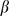
9 strand. These residues were mainly in the 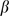
7–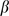
9 zone. We also noticed the presence of conserved charged residues at the end of the L34 loop and in the first half of the L57 loop.

Other positions also appeared to be highly conserved such as G19, L34 and A78, but were not common properties to all sHSPs. Moreover, many were group-specific as shown in the logo representation of animals, plants, bacA and bacB (Fig. 4B to E). For example, G19, L34 and A78 residues were not predominant in animals. Instead, specific residues or motifs such as F11, D-V-X-X-F-X-P, K28 in the 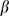
3–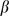
4 zone or G-K-H-E-E-R/K in the very beginning of the L57 loop, Y65 (rather than F65) in 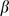
7 or a serine-rich motif in 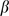
8 (S-S/T-L-S) were observed in this group.

The G19 residue actually belongs to the P-G doublet involved in the dimer interface of two subunits (for HSP 16.5, HSP 16.9 and HSP A structures). In a study on 344 sHSPs, Fu *et al.* [36] showed that the P-G doublet is highly conserved in most of the non-animal sHSPs. The L34 loop (annotated in Fig. 4 in positions 17 to 24) generally starts with an aliphatic residue and ends with two (usually negatively) charged residues. As in ref. [36], the P-G doublet is surrounded by hydrophobic residues (Fig. 4). In animals, this motif was missing and was replaced with charged amino acids [36]. The hydrophobic residue in position 20 was usually a F (as for bacA) and a P residue was found in position 22 (Fig. 4B). The plant and bacB groups clearly showed the P-G doublet (Fig. 4 C and E), whereas the bacA group showed a highly conserved A-G doublet, with neighboring residues being mainly V and F (Fig. 4D). Although we used a different methodology and had ten times as many sequences, our results are perfectly in line with the findings of Fu *et al.* [36]. Furthermore, the bacA group showed a unique, intermediate feature. Based on their highly conserved A-G motif, bacA sHSPs were similar to plants and bacB. However, the P-to-A substitution in the original P-G doublet was unique and exclusive (97% of bacA sequences possessed the A-G doublet compared to 1% for P-G). Finally, hydrophobic neighboring residues of the doublet were mainly V and F, as for animals. In addition, sequences in animals and bacA shared several other common residues such as H53, G55, R59 in the second half of the L57 loop.

In bacA, the sequence logo representation showed several well-conserved motifs in 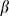
2 (Y-N-I/V-E), 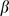
3 and the L34 loop (Y-R-I-X-X-A-V/L-A-G-F), and in the beginning of L78 loop (L-A-D/E). In contrast to other groups, the sequence logo revealed a very well-conserved second half of L57 loop, a shortest L78 loop (no residue detected in positions 75 and 76) and, a specific 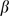
9 end (D/E-L-X). BacA sequences were also characterized by the absence of proline residues that were usually conserved in other groups (positions 18, 68, 91) and were replaced by an alanine (at positions 18 and 68). Conserved alanine residues are specific to this group, such as A16 (replaced by a negatively charged D/E residue in other groups) or A57. Sequences in plants often shared several characteristics with bacB (such as residues D2 and E5 in 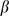
2, motifs G-E-R and R-X-E-R-X-X-G in the L57 loop) or, more generally, with non-animal sequences (such as P/A-G doublet in the L34 loop, L34 in 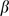
5 or A78 in 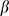
8).

To summarize, the ACD region is the hallmark of the sHSP family, both from a sequence and a structural points of view. We used this feature to fetch sHSP sequences through the UniProt databank. Nevertheless, we revealed that ACD also has unique features that are specific to some groups.

### The C-terminal Anchoring Module (CAM)

**Figure 5:**
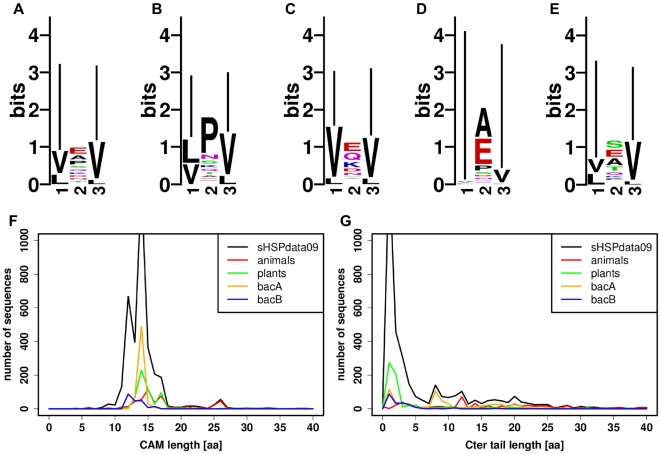
Logo representation of the I/V/L-X-I/V/L motif and length distributions of CAM and Cter tail. Logos are displayed for (A) sHSPdata09, (B) animals, (C) plants, (D) bacA and (E) bacB. The length distributions of CAM (F) and Cter tail (G) are computed for sHSPdata09 (in black), animals (in red), plants (in green), bacA (in orange) and bacB (in blue). The tops of the sHSPdata09 length distributions are truncated for reasons of clarity. Sequence lengths correspond to a number of amino acids (aa).

A conserved motif located in the C-terminal region [15] is directly involved in the oligomerization process [45] and is interacted with the hydrophobic groove of ACD in HSP 16.5 and HSP 16.9 structures [16], [17]. This motif is also referenced in the literature as I-X-I [15], I/V-X-I [46], I-X-I/V [17] or I/V-X-I/V [18] patterns. The I/V-X-I/V motif was found in 90% of sHSPdata09. As I and V are aliphatic residues, we also searched for an extended I/V/L-X-I/V/L motif that we found in 96% of our dataset. This motif, found in a polar region, appeared to be an important characteristic of the sHSP family. The logo representation of the I/V/L-X-I/V/L motif in Fig. 5A shows the predominance of I residues at both extremities of the motif. However, at the central position, we could not see a clear signal of most frequent residues. We therefore give the logo representation of the motif for all groups separately (Fig. 5B to E). The central position for animals was usually a proline, which was not found in other groups (except for bacA, after A and E residues), and sometimes a glutamate (often observed in other groups). The bacA group showed predominant A/E residues in the central position and I in the first and last positions.

The strong presence of the I/V/L-X-I/V/L motif in the C-terminal region and its anchoring role in sHSP oligomers led us to study the length of the fragment from the end of ACD to the end of the motif that we defined as the C-terminal Anchoring Module (CAM). The last part of the C-terminal region, located after CAM was called the C-terminal tail (Cter tail). For sHSPdata09, CAM had an average length of 14 residues and 85% of the sequences with the motif had a CAM length of 14
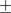
3 residues (Fig. 5F). All studied groups showed a similar CAM length distribution. However, drosophila and tick sHSPs presented a specific CAM length of 25–26 residues that appeared to be a strong characteristics of these two species. The Cter tail appeared rather short with 65% of sequences having a Cter tail smaller than 5 residues (Fig. 5G). Plants and bacB shared this feature, whereas animals showed an average Cter tail length of 10 residues and bacA had either short or long Cter tails.

## Discussion

### Detection of sHSPs with the ACDP09 profile

In the present work, we built an HMM profile dedicated to the identification and the structural annotation of protein sequences belonging to the sHSP family. The originality of our procedure lies in combining an initial profile solely based on all available structural data with an iterative procedure. In theory, this approach could be easily transposed to other protein families with low sequence identity, few available structures but with a strong structural pattern.

Our profile was initially built on a multiple sequence/structure alignment of 11 structurally well-characterized ACDs. From a structural standpoint, we defined ACD as the region delimited from the 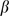
2 strand to the 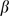
9 strand. The ACDP09 profile fetched 4478 sequences from UniProt that were consistent with current sHSP annotations. These sequences were all characterized by the presence of one complete ACD and, after curation, 3787 proteins were analyzed as the sHSPdata09 dataset. To our knowledge, this was the first time that an exhaustive study of more than one thousand sHSPs was conducted.

### Architecture of the monomer and common specificities to the sHSP family

Based on ACD detection, we deduced the architecture of sHSP monomers. We clearly delineated the three structural regions (N-terminal, ACD, C-terminal) in each sequence.

As reported in the literature, the N-terminal region is the most heterogeneous region in terms of length and sequence, making it difficult to extract any general properties of sHSPs [8], [15], [20].

The analysis of ACD, known as the signature of the family, revealed strong specificities shared by the sHSPdata09 sequences identified in this study. We found that the length of ACD is 90
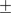
10 residues and is highly dependent on the L57 loop length whereas the average length of the 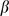
2–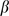
5 and 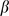
7–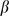
9 zones is conserved. The logo representation of ACD, where positions refer to the ACDP09 profile, displays residues or motifs that are very conserved in sHSPs. Interestingly, these residues are mainly localized along the 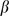
7–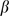
9 zone (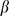
7 strand, L78 loop, L89 loop/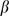
9 strand). The motif identified in the 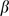
7 strand contains the arginine residue associated with human pathologies [4], which is clearly conserved even on a set of 3787 sHSP sequences. Other residues observed at this position in much lower frequencies are L, K and Q. In the 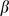
2–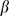
5 zone, we found alternating hydrophobic residues specific to 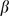 strands (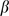
4 and 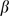
5) and a terminating G/A residue. At last, the beginning of the L57 loop is marked by charged residues followed by some isolated conserved residues.

In the C-terminal region, 96% of the sHSPdata09 sequences have the motif I/V/L-X-I/V/L, confirming that this motif is a common characteristic of sHSPs. Interestingly, the distance from the end of ACD to the end of the motif is well conserved. We named this fragment the C-terminal Anchoring Module (CAM). This result demonstrates that CAM has a more constrained length than what is commonly supposed on the localization of the motif in the C-terminal region [14]–[16]. Consequently, the variability of this region is mainly due to the Cter tail (residues following CAM). The existence of CAM supports the critical role of the I/V/L-X-I/V/L motif in the stabilization of sHSP assemblies [17], [23], as illustrated by HSP 16.5 and HSP 16.9 oligomeric structures.

### Divergence between sHSP groups

In this study, we focused on homogeneous groups having a sufficient number of sequences (at least 200). Four groups were considered: animals, plants, bacA and bacB. Detailed analyses highlighted group specificities that could constitute a first step toward a classification procedure (as illustrated in ref. [35]). Three distinct peaks in the ACD length distribution are associated with sequences in animals (83 residues), bacA (86 residues) and plants (90 residues). These characteristic ACD lengths are directly related to the L57 loop lengths, where animals are mainly associated with the shortest L57 loop (13 residues). The logo representations built for each group illustrate specificities in terms of structural zones or residues. Sequences in animals are distinguished from non-animals in structural elements involved in function or in the oligomerization process. Animal sequences specifically show a poorly conserved 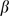
2 strand, and conserved residues/motifs in the 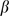
3 strand, the L34 loop, the 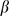
4 strand, the very beginning of the L57 loop and in the 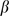
7 and 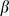
8 strands. Interestingly, the 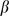
2 strand is not always seen in mammalian structures [22], but is involved in the dimerization of non-mammalian structures (*i.e.* HSP 16.5, HSP 16.9 and HSP A). The fragment from the 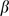
3 strand to the 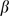
4 strand is functionally relevant because associated with substrate binding in mammalian 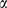
A and 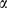
B crystallins [24], [37], [47]. Finally, the 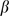
7 and 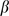
8 strands are involved in the association of monomers [22] or dimers [16], [17], respectively. Moreover, animals share several residues with bacA (in the L34 loop and in the second half of the L57 loop), whereas plants share several characteristics with bacB (in the 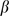
2 strand and in the L57 loop). BacA sequences display their own characteristics with specific conserved motifs (in the 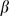
2, 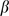
3 and 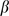
9 strands and in the second half of the L57 loop), the shortest L78 loop of all groups and a different usage of proline and alanine amino acids. The conserved proline residues in other groups are often replaced by an alanine in bacA (the A-G and L-A doublets in the L34 and L78 loops, respectively), and conserved alanine residues in bacA are not seen in other groups (*e.g.* last residue in the 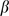
3 strand).

An analysis of the C-terminal region also revealed group specificities. A strong group preference for the X residue in the I/V/L-X-I/V/L motif was observed: animals and bacA preferentially show I/L/V-P-I/V/L and I-A/E-I motifs respectively. To go further, a P residue at the central position is a signature of animals whereas an E residue is related to non-animals. The Cter tail (zone following the I/V/L-X-I/V/L motif) is generally very short in plants and bacB (less than three residues on average), but can be significantly longer in animals (10 residues on average) and bacA (8 residues). Indeed, this Cter tail has been described as a very flexible zone in NMR studies of 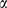
A and 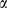
B crystallins [48].

In conclusion, this work provides new insights on the structural organization of sHSP monomers. Structural elements identified as essential to the oligomerization process are associated with specific sequence properties in each group. Particularly, animals are characterized by a short L57 loop, a poorly conserved 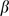
2 strand and the absence of the P-G doublet in the L34 loop. All these structural elements are known, however, to be involved in dimer formation in the non-animal HSP 16.9, HSP 16.5 and HSP A structures. Differences between animal and non-animal sHSPs are confirmed by the recently published crystallographic structures of two mammalian sHSPs (and also by the worm TSP 36 structure) that present a dimer organization distinct from the previous known dimers found in plant, archaea or bacteria [22]. Finally, we show the relevance of the iterative HMM approach based on structural data, learned on sequences but aimed toward elucidating structural/functional properties of sHSPs.

## Materials and Methods

### Selection of the initial dataset and structural delineation of ACD

Seven ACDs are available at atomic resolution from crystallographic structures of sHSPs: HSP 16.5 (PDB 1SHS [16], UniProt Q5773) from the archae *Methanococcus jannaschii*, HSP 16.9 (PDB 1GME [17], UniProt Q41560) from the plant *Triticum aestivum*, HSP A (PDB 3GLA [21], UniProt Q8PNC2) from the bacterium *Xanthomonas axonopodis pv. Citri*, TSP 36 from the worm *Taenia saginata* (PDB 2BOL [18], UniProt Q7YZT0) with 2 ACD, HSP 20/HSP B6 (PDB 2WJ5 [22], UniProt P97541) from the mammal *Rattus norvegicus* and 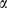
B crystallin/HSP B5 (PDB 2WJ7 [22], UniProt P02511) from *Homo sapiens*. The structures deposited in the Protein Data Bank are either the entire complex (24-mer for HSP 16.5, 12-mer for HSP 16.9, 4-mer for TSP 36) or just dimers (for HSP A, HSP 20 and 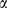
B crystallin). The secondary structures from 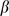
2 to 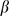
9 are assigned from dssp [49], and confirmed by the literature and by pairwise structural comparisons. These structures were supplemented with four sHSP sequences structurally well-annotated with ACD delineation clearly indicated: human 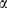
A crystallin (UniProt P02489) (annotated from site-directed spin labeling [50] and conformational studies on a wildtype and mutants [51]), HSP 16.3 from the bacterium *Mycobacterium tuberculosis* (UniProt P0A5B7) (annotated from mass spectrometry and electron microscopy [52], multiple sequence alignment and structural characterization [36]), HSP 26 from the yeast *Saccharomyces cerevisiae* (UniProt P15992) (studied with cryoelectron microscopy [53]), and IbpA (UniProt P0C054) from the bacteria *Escherichia coli* (often included in multiple sequence alignment analysis [3], [15], [36], [37]).

### Sequence/structure multiple alignment

We designed a robust alignment procedure based on a mixed structural and sequence multiple alignment of manually delineated ACD regions. The multiple alignment was created using the 3DCoffee [39] web server using advanced options (slow_pair, sap_pair, fugue_pair). Our ACD reference set had seven ACDs extracted from 3D structures and four extracted from sequences. We carefully inspected the alignment to verify its consistency with the literature. More specifically, we manually checked that 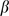 sheet assignment and known motifs are aligned (*i.e.* the P-G doublet in non-animal sequences [36], the well-conserved arginine residue in the 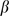
7 strand [25], [40]). This high-quality alignment was subsequently used to create the initial Hidden Markov Model (HMM) profile with HMMER (version 2.3.2) [54], [55] using standard parameters.

### Iterative Hidden Markov Model profile generation

Protein sequences identified as sHSPs were found in the UniProt [29] databank (release 15.9 of October 13, 2009) that contains more than 10 million protein sequences. We made no distinction between reviewed (manually annotated, termed SwissProt) and unreviewed (automatically annotated, termed TrEMBL) proteins.

An iterative procedure (iHMMER) was established to perform a specific and sensitive search of the UniProt databank. The full process can be described by the following steps:

1. Generate the initial profile from the structural alignment.
2. Scan the UniProt databank for sHSPs with the profile and a given E-value threshold (start at 10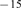).
3. Filter (no ACD length greater than 100 residues) and extract detected ACD.
4. Align selected ACD sequences in the profile.
5. Generate a new profile from the alignment.
6. Calibrate the profile.
7. Increase by one order of magnitude the E-value threshold and return to step 2 until a threshold E-value of 10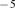 has been reached.

The iHMMER procedure converged quickly to roughly 4000 sequences. After 10 iterations, a final step selected sequences with only one ACD and an ACD detection E-values of 10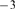 or less.

Fetched sequences were then compared to sHSP sequences labeled within UniProt with at least one of the following annotations: family:“small heat shock protein, family:“HSP20”, “prosite PS01031” and “pfam PF0011”. The first two are common sHSP names, whereas the last two are profiles deposited in the PROSITE and the Pfam databanks, respectively.

### Manual curation, sequence refinement, region analyses and sequence slicing

Sequences with a single ACD were selected based on their UniProt annotations (no fragment and protein existence level less or equal to 3) and then divided into three regions (N-terminal, ACD and C-terminal) from their ACD delineation. Following the classification performed in the work of Fu and Chang [36], sHSPs were grouped based on their taxonomic origin into archaea, fungi, plants, animals and other eukaryotes (termed other). Based on pairwise global alignments, bacterial sHSPs were classified as class A bacteria (bacA), class B bacteria (bacB) [38] or neither class A or B (bacOther). From the positioning of the L57 loop in the profile, identified ACDs were then subdivided into the 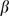
2–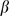
5 zone (composed of the 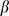
2, 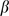
3, 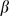
4 and 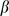
5 strands), the L57 loop and the 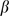
7–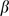
9 zone (composed of the 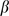
7, 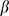
8 and 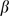
9 strands). These three zones were analyzed in terms of length and hydropathy score (defined as the sum of the Kyte and Doolittle hydropathy indexes [43] of a given zone divided by its length). We also looked for the I/V-X-I/V [18] and I/V/L-X-I/V/L motifs in the C-terminal region with the help of regular expressions.

A sequence logo representation of the predominant amino acids was built from residues that matched profile positions. We use a modified version of WebLogo [56], [57] where gaps are explicitly taken into account as a 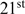 amino acid but not drawn in the logo.

All statistical analysis and graphics were done using R [58].

## Supporting Information

Dataset S1 List of the 3787 sequences constituting the sHSPdata09 dataset. First column is the UniProt accession, second column is the corresponding group and third column is the length of the detected ACD.
(0.10 MB PDF)
