# Supplementary material for: Detection and Architecture of Small Heat Shock Protein Monomers
Source: PLoS One. 2010 Apr 7;5(4):e9990. doi: 10.1371/journal.pone.0009990 (PMC2850924; doi:10.1371/journal.pone.0009990)
Supplement: Dataset S1 — List of the 3787 sequences constituting the sHSPdata09 dataset. First column is the UniProt accession, second column is the corresponding group and third column is the length of the detected ACD. (0.10 MB PDF) [file pone.0009990.s001.pdf]

accession	group	ACD_size
B9SA05	plant	91
C0QVM3	bacOther	88
O01718	animal	101
A1E463	plant	90
B0EHH3	other	87
A0K2I6	bacOther	90
C1C473	animal	83
Q1W289	animal	83
B6T6N6	plant	90
Q3STL4	bacOther	86
Q8DDJ3	bacA	85
Q061S4	bacOther	89
Q99RP2	bacOther	86
Q16S85	animal	91
B7P7F7	animal	84
Q97W19	archae	75
Q5KY05	bacOther	90
Q3M3M7	bacOther	89
B9ZK50	bacOther	89
B5YX93	bacA	86
P81958	bacOther	89
C3MMU1	archae	87
Q3BWG0	bacOther	89
A8N7T4	fungi	91
Q884C5	bacA	87
Q6G5C1	bacA	88
A8DVB3	animal	87
C2UUV0	bacOther	92
Q8YEU3	bacA	89
B7CUQ8	bacOther	90
B7CUQ9	bacOther	88
B9AWU5	bacOther	107
C1ZPF2	bacOther	88
A6V2U7	bacA	87
B7P7F8	animal	113
C4J643	plant	82
A0R3V7	bacOther	88
Q9DEU9	animal	83
A5BWS0	plant	104
A1SBM6	bacOther	87
A9HCX4	bacA	87
Q4FXF7	other	90
A3WKG4	bacA	86
O86110	bacA	85
C0ACF3	bacOther	88
C0ACF2	bacOther	81
B4B9Q4	bacOther	89
B6SRT5	plant	91
Q7NYU5	bacB	90
A4AV49	bacOther	89
Q03DQ8	bacOther	90
C1XLB0	bacOther	89
Q8L8R5	plant	95
Q73UU8	bacOther	87
Q2LCS7	animal	83
C2CEW0	bacOther	90
B6TDB5	plant	90
Q3E986	plant	79
A2WKD5	plant	90
A2WKD4	plant	90
A2WKD7	plant	90
Q21BA2	bacA	88
B4PCB3	animal	83
A2WKD3	plant	91
A2WKD2	plant	93
Q1BGH5	bacB	92
Q1BGH4	bacOther	88
B2H512	bacOther	90
B7CDM9	bacB	91
A6SMR3	fungi	98
B9RV59	plant	90
A1KAK3	bacOther	90
B2JMU8	bacOther	88
B2JMU9	bacOther	91
Q8TZC8	archae	86

accession	group	ACD_size
B2AV59	fungi	158
Q5ZTH1	bacA	87
Q4WYW9	fungi	124
Q3R3S4	bacOther	89
A8NZF2	fungi	120
Q8Z2L8	bacA	86
B6TD78	plant	97
A9RDF3	plant	80
C0PTB9	plant	92
Q86GU1	animal	135
A5BAN4	plant	90
B5YBV6	bacOther	89
B9SIR7	plant	91
A0YRM5	bacOther	89
A5JBH0	bacOther	90
Q82Q29	bacOther	89
A0JN13	animal	83
P15992	fungi	95
Q5LV07	bacA	86
P15990	animal	83
B8AJJ8	plant	99
C1Y3Q3	bacOther	87
C0QLP0	bacOther	88
C0QLP1	bacOther	86
B4ARN0	bacB	87
Q66KZ0	animal	83
B4IXA0	animal	91
B4IXA1	animal	97
Q647I7	other	94
Q3AGM9	bacB	89
C0ECQ3	bacB	90
Q2C1M0	bacA	85
C0PJF1	plant	80
B6AYF6	bacA	86
A8P232	animal	85
B5JL23	bacOther	85
B5JL22	bacOther	89
A9NKX8	plant	90
Q74FR8	bacOther	89
B1QS45	bacOther	88
B5NU19	bacOther	88
A6E187	bacA	86
A9NPB3	plant	90
Q5U8W2	animal	83
Q3B508	bacOther	73
O13225	animal	87
C3G2J2	bacOther	90
Q4RYB9	animal	83
B3RCY4	bacOther	88
B1Z7U3	bacA	87
B1C2K1	bacOther	91
B6BCR6	bacA	86
B6W2V6	bacOther	92
Q1YK58	bacA	88
A0KDX0	bacB	92
B7R1Y6	archae	89
A0LHQ8	bacOther	91
Q8YDY2	bacA	88
Q54W43	other	143
A9VAH3	other	133
C0UVN5	bacOther	89
A6EY58	bacOther	89
A2X0F6	plant	90
A9BVF2	bacOther	74
A9HFK2	bacOther	87
B3Q5Y9	bacA	90
Q49ZX4	bacOther	87
Q49ZX5	bacOther	90
B7AK63	bacOther	97
C1B6Y2	bacOther	87
A4BDK6	bacA	85
A4BDK7	bacOther	89
A7YR80	bacB	87
B6XIB9	bacOther	88
B3X8S0	bacA	86

accession	group	ACD_size
C4ANX2	bacOther	88
Q16S84	animal	83
A8EX24	bacOther	89
B9S3B4	plant	90
C4JZE7	fungi	146
B0TJF1	bacA	86
B9S3B3	plant	90
B9S3B2	plant	90
A7P4P7	plant	90
B8FLL8	bacOther	88
A1CE36	fungi	139
B9S1R8	plant	90
B1L7F8	archae	69
C2WLY4	bacOther	90
B8FLL7	bacOther	88
A7P4P8	plant	90
A7P4P9	plant	90
A6EJW7	bacOther	89
Q40978	plant	91
C0NER7	fungi	101
Q1KS37	animal	100
Q16JF9	animal	83
B3M6E9	animal	92
Q7VQU7	bacA	87
A5G8D6	bacOther	89
Q72QA2	bacOther	89
C6T2L4	plant	90
Q6L891	fungi	111
Q72QA1	bacOther	87
P82147	animal	93
B4NLH7	animal	91
B3M6E6	animal	116
Q9N350	animal	83
Q1H248	bacOther	89
Q7G754	plant	100
C4DED9	bacOther	89
B2FH35	plant	90
A7VRF1	bacOther	89
B2FH37	plant	90
B2FH36	plant	90
C1DSQ8	bacA	91
B2FH33	plant	90
A0QE71	bacOther	87
A3CWD4	archae	90
Q2LVT0	bacOther	89
A5G1U1	bacA	86
Q16JG7	animal	83
B2FH39	plant	90
B2FH38	plant	90
B5XT61	bacA	86
Q0W4L5	archae	90
A8IXM7	plant	96
Q1H3V8	bacA	88
A5A8U4	plant	87
Q2L3S1	plant	101
Q7G566	plant	85
C3DJ87	bacOther	91
Q4XZX9	other	96
Q5V154	archae	90
B4MY14	animal	83
A5IVG8	bacOther	86
B5CA47	bacA	86
A9NPT1	plant	92
B5XT60	bacA	86
B5X8F0	animal	87
C1ZL26	bacOther	89
C1ZL27	bacOther	88
B4HKN7	animal	116
B4HKN9	animal	92
C1CX79	bacOther	88
C2D8E4	bacOther	88
Q3JNQ5	bacOther	88
Q3JNQ4	bacB	91
Q8XGW7	bacA	86
Q0KKB1	animal	83

Q92VA9	bacA	85
Q0KKB2	animal	83
B4IYG9	animal	93
A3WW64	bacA	89
B3ZA72	bacOther	91
B6BVP0	bacOther	95
B4UXB2	bacOther	89
B5RM03	bacOther	89
B7GHV5	bacOther	90
B7HQ56	bacOther	90
B6T649	plant	84
A4XBV1	bacOther	87
A2P574	bacA	85
B1G0A9	bacOther	88
Q7XUW5	plant	94
A9W7W4	bacB	89
A4XL56	bacB	88
A1ZM02	bacOther	88
Q2JAZ8	bacOther	87
C3SLR7	bacA	86
A4XVW5	bacA	87
A5K0L0	other	96
C1CV16	bacOther	84
A9DUT6	bacA	86
B7U6N2	other	95
A5BGC0	plant	90
B9HKJ2	plant	80
B1Y2X7	bacOther	91
A6DR96	bacB	88
A6DR97	bacOther	84
Q0S231	bacOther	88
A1VDV9	bacOther	81
B0G2I5	bacOther	91
B4LGS7	animal	118
Q29F95	animal	84
Q2RFK2	bacOther	90
Q29F97	animal	98
Q29F96	animal	121
Q13ZV9	bacOther	88
Q13ZV8	bacOther	89
Q29F93	animal	93
Q29F92	animal	83
B6TPF4	plant	90
B5IAD9	archae	88
Q29F98	animal	95
Q5MGN8	animal	83
Q6WHC0	plant	90
A3P9W2	bacB	90
Q5FNB9	bacA	86
Q8R745	bacOther	89
B8EMF3	bacOther	88
A9MG56	bacOther	88
A8WLE6	animal	83
Q943Q3	plant	93
B2BY13	plant	90
B7ZEP9	plant	86
A9A2S7	archae	90
A8H3U4	bacA	86
A4CMN6	bacOther	89
A0QM89	bacB	90
C4G297	bacOther	92
B7ZEP5	plant	84
B7ZEP6	plant	82
B3R4R2	bacOther	88
B3R4R1	bacOther	91
Q1WLA9	bacOther	73
Q31KI8	bacOther	89
A8LJ34	bacB	89
A2SHH7	bacOther	89
B1UIZ7	bacOther	90
A7VJH1	plant	96
C4CMU2	bacOther	97
B6TXB5	plant	90
A4ABG0	bacA	86
Q7A030	bacOther	86
B8I9W5	bacA	86
A6TI56	bacOther	89
A4T3Y7	bacB	93
B9SI99	plant	79
Q20363	animal	82
C3SLR2	bacA	86
B1ZS67	bacOther	88
Q98H48	bacA	88
B0WEQ9	animal	88
B0WEQ8	animal	88
B9MYM3	plant	80
A7GBM2	bacOther	88
A5ETZ1	bacOther	78
B0WEQ7	animal	83
B0WEQ6	animal	83
B0WEQ5	animal	110
B0WEQ4	animal	83
C3HI16	bacOther	91
A5CX04	bacOther	74
B5Y472	other	81
B9ZP65	bacOther	89
Q7NH34	bacOther	88
C4L9A6	bacOther	87
A8TXB9	bacA	87
B9RQ27	plant	90
B7CDN0	bacB	91
A3CX12	archae	90
A5ETZ0	bacOther	88
C1WI58	bacOther	89
C3GIF0	bacOther	91
A2X2G6	plant	97
B2PZR1	bacOther	88
A0RDN1	bacOther	91
Q1ATM9	bacOther	89
Q13CS9	bacA	88
B3J2G9	bacOther	91
A6X6X5	bacA	88
Q0TB21	bacA	86
Q2PPK4	animal	83
Q2PPK2	animal	83
Q2PPK3	animal	83
Q2PPK0	animal	83
Q569M7	animal	83
C2KDN8	bacOther	90
A2ZD05	plant	98
A8YEZ9	bacB	84
C6TEC9	plant	100
Q0RZ94	bacOther	87
A6TFY9	bacA	86
Q47Y72	bacA	94
C2XBA8	bacOther	90
Q39HR5	bacOther	88
Q39HR4	bacOther	91
Q75BF9	fungi	95
C3X4S9	bacOther	90
O30851	bacOther	90
Q4F877	animal	83
A1SV28	bacOther	89
A8Y0T5	animal	83
A5BP28	plant	93
P0C056	bacA	86
A8Q2A9	animal	86
C1BPW1	animal	86
A8MBU8	archae	88
B4LGS8	animal	96
B4LGS9	animal	90
B4LGS6	animal	84
C3EKA2	bacOther	92
B5NIU8	bacOther	88
Q0G4R1	bacA	88
B7QY10	bacA	86
B0SY21	bacA	86
C4CZ90	bacOther	92
A3JND0	bacA	85
C0C4C3	bacOther	91
A9NU13	plant	90
Q41560	plant	90
B0AQN0	bacOther	91
A4HHP4	other	90
Q41568	plant	92
B5JW68	bacOther	89
Q9W6T3	animal	83
A5K5U4	other	95
Q9BMF0	other	95
Q6TAA6	bacA	87
A1BIT0	bacOther	89
Q2JYH4	bacOther	85
A8XDE7	animal	83
Q16JG0	animal	83
Q747S3	bacOther	88
B3L1E1	other	95
B8GPA9	bacOther	89
C1ME18	bacA	86
Q1E147	fungi	145
A1F590	bacA	85
B1MUD3	animal	83
B4UZ10	bacOther	89
C4KV04	bacOther	88
C4KV05	bacOther	90
A4LA97	plant	91
C3E2X3	bacOther	90
B5EPP8	bacOther	87
A9G450	bacA	86
Q3SQR8	bacB	86
Q3SQR9	bacA	85
C3KJP8	animal	83
Q5H4F8	bacOther	89
A1E385	animal	130
C0S1A4	fungi	131
A5IDZ2	bacOther	89
Q3R9G4	bacOther	89
Q21Y84	bacB	91
Q21KB7	bacA	86
A7P4Q5	plant	90
A7P4Q4	plant	90
A7P4Q7	plant	90
A7P4Q6	plant	90
B9TLQ5	plant	102
A7P4Q2	plant	90
Q9HSD8	archae	90
A7P4Q8	plant	90
B5FMC9	bacA	86
B3M6F4	animal	94
C7BVK7	animal	82
C1DQE3	bacA	87
B3M6F0	animal	84
A5GBZ4	bacOther	89
A4MF28	bacB	91
B3M6F3	animal	99
A4MF27	bacOther	88
Q8MZU6	other	84
B2A6N9	bacOther	92
B5XQZ7	bacOther	90
B7P624	animal	84
B9KNG2	bacA	86
B9BSJ8	bacOther	85
B6FG30	bacA	86
Q4P7N2	fungi	141
B1LL12	bacA	86
B7AVH7	bacOther	92
B1LL11	bacA	86
Q92M33	bacA	87
A4SCX8	bacOther	89
C0YKG7	bacOther	89
B8CY41	bacOther	89
Q8LBT4	plant	79
A8A6E7	bacA	86
A4GAF6	bacA	86
C1DEN7	bacOther	89
B1KAX5	bacOther	88
B1KAX4	bacB	92
C3L3U7	bacOther	91
A1E2A9	plant	90

C3J648	bacOther	88	Q551G1	other	147	Q0RZ81	bacOther	89
A5AQ47	plant	90	B7ZEQ0	plant	94	B2I5Z4	bacOther	89
Q07160	animal	82	A9SQV2	plant	90	P46732	bacOther	87
B1JZ10	bacOther	91	Q2QJL7	bacA	86	O31288	bacOther	93
C2MUP3	bacOther	90	Q54I91	other	93	Q2LPJ5	bacOther	88
C1XR11	bacOther	88	Q88HP0	bacOther	88	Q2LPJ6	bacOther	89
A9NL04	plant	82	C0XDX5	bacOther	90	Q9M6R2	plant	90
Q0KBR3	bacOther	91	B9RQ29	plant	79	A3MX90	archae	75
A3N813	bacOther	90	A3W4R6	bacA	86	B1N203	bacB	91
A3N812	bacOther	88	Q39818	plant	82	A7QCE2	plant	90
Q5ZTH0	bacOther	89	Q39819	plant	95	B7RD54	bacOther	89
A6UIE4	bacA	85	A2SHG2	bacOther	88	A8KGY5	bacOther	90
A6ZYC9	fungi	93	C6TMV0	plant	94	B4JCI5	animal	91
C1BJV2	animal	83	B9R949	plant	79	A7AVD6	other	86
B1MV71	animal	94	B5Y3Y4	other	84	B6R764	bacA	86
Q9ZP25	plant	83	Q9GSB6	animal	83	Q1J023	bacOther	84
Q7Q723	animal	83	Q0I6F9	bacOther	103	Q17849	animal	135
Q7Q724	animal	83	Q5B7C5	fungi	126	A9B5N0	bacOther	89
Q54VP4	other	89	B3WBX7	bacOther	90	A0LR75	bacOther	88
Q2G6S0	bacA	85	A6PQY9	bacOther	89	Q75F56	fungi	95
Q19227	animal	83	B9GVE8	plant	82	B7Y7J2	bacOther	90
B6U3J2	plant	93	A6UT17	archae	90	Q88HN9	bacOther	91
Q79K03	bacOther	90	B8HJQ7	bacOther	89	B3C8N3	bacOther	92
A0K166	bacB	92	B3NCF1	animal	99	A5YRZ1	plant	90
C0PSE6	plant	86	C2FDJ8	bacOther	90	B9NTK2	bacA	86
B8HWL6	bacOther	89	B4EI89	bacOther	90	B3H5Q4	plant	91
A9ZZC0	bacA	86	B1YMT1	bacOther	91	C1SIE4	bacOther	89
C2CYT6	bacOther	90	B1YMT0	bacOther	88	B6B2L1	bacA	86
C2R7P9	bacOther	90	C1XR05	bacOther	69	C7BVQ8	animal	90
Q7CPF1	bacA	86	A5EHH6	bacB	89	O82825	bacOther	89
A0QPJ9	bacB	90	A3KWF7	bacA	87	A9CKC3	bacA	90
Q89H22	bacOther	87	Q92Z87	bacB	89	B5Q694	bacA	86
Q2S165	bacB	89	A9UEZ0	animal	116	B3QF80	bacA	87
B8NXF5	fungi	90	P15991	animal	83	C2QSK3	bacOther	92
A5CVD9	other	90	B8DPI1	bacOther	81	C2LFA5	bacOther	88
A1VLW0	bacOther	91	A8GMS9	bacOther	85	B8BH26	plant	84
C0BJE5	bacOther	89	Q6N5S5	bacOther	87	Q52L78	animal	83
Q1YY52	bacA	87	B2TGA3	bacOther	91	A0QG70	bacOther	87
B2N6C3	bacA	86	A0T073	animal	83	A5L6E9	bacA	85
B1EZ46	bacOther	91	A9UEZ2	animal	96	A0QG72	bacOther	87
C3PMX1	bacOther	85	A9CUG3	bacOther	89	B3X8S1	bacA	86
B2II78	bacOther	89	Q60HG8	animal	83	A8J2Z3	plant	189
A9FKI0	bacOther	89	B9XB80	bacOther	93	C2T105	bacOther	90
B9GTI4	plant	94	A9UEZ3	animal	84	B6EGS9	bacA	86
B8N2K7	fungi	143	B9HAX6	plant	72	C6DGL0	bacA	86
Q11YJ1	bacOther	91	C3C1V4	bacOther	92	Q7G755	plant	85
A1RXC6	archae	72	B6V9W9	other	95	A5UPT0	bacB	89
Q9ZW31	plant	90	B9H8R6	plant	85	B5XD67	animal	80
Q1MI33	bacOther	89	Q39929	plant	90	A6L7D4	bacOther	96
A5BGB5	plant	90	Q5RAB0	animal	142	B1Z4J0	bacOther	73
Q1AY86	bacOther	87	Q3SJ47	bacOther	88	B1Y2X8	bacOther	88
A5BGB8	plant	91	Q8IQW5	animal	124	B7JAY7	bacOther	89
B9ZHA4	archae	90	C4R9G1	fungi	97	A8I6R6	plant	91
B9Z998	archae	73	A9EIB4	bacA	86	Q4L8Q4	bacOther	86
C0C840	bacOther	91	Q711Q1	animal	82	A3TTS9	bacA	86
Q62H41	bacOther	88	A7K7F4	bacOther	107	B7Q2C4	animal	83
B3PDH2	bacA	86	C3GIG1	bacOther	90	Q9SE12	plant	86
Q2Y9U8	bacOther	89	B2AYV7	fungi	100	Q9SE11	plant	86
C4I0D5	bacA	86	C3B351	bacOther	92	B7MGA7	bacA	86
Q8FXL4	bacA	88	B6KN75	other	89	A2ZNZ7	plant	91
C2VT96	bacOther	80	A5TMA0	bacOther	88	Q7JP52	animal	83
B0SYI5	bacB	89	B0CVA2	fungi	120	P68405	animal	83
B9QBE4	other	95	Q8Z9V5	bacA	86	P68406	animal	83
Q13ZU0	bacOther	90	A8A6E6	bacA	86	B0K455	bacOther	89
Q2FLP2	archae	89	B3J2F9	bacOther	90	A9MJW8	bacA	86
B5FK56	bacOther	88	Q9ZSR5	plant	92	A9MJW9	bacA	86
Q18J73	archae	90	Q8EMZ6	bacOther	89	A4EB60	bacOther	88
C1C1Z3	animal	86	Q6MJP8	bacB	86	P72977	bacOther	89
C0FHS9	bacOther	91	Q20660	animal	83	Q39QP6	bacOther	88
Q3IUT4	archae	73	A3X699	bacA	86	A4BWG1	bacOther	73
A6B5Z8	bacA	85	O03993	plant	87	B3JHD1	bacOther	90
C4CRI8	bacOther	89	B7QNN7	animal	84	C1RL68	bacOther	88
B3IKH3	bacA	86	B3EHR2	bacOther	89	Q29EN7	animal	83
B3YT35	bacOther	90	Q1BFF6	bacB	90	Q8W541	plant	90
B7NQY6	bacA	86	B9ST51	plant	94	Q5UZZ7	archae	85
Q8L543	plant	91	Q12988	animal	102	Q17MD5	animal	84

Q17MD6	animal	123
Q5MGP0	animal	83
B9I5V3	plant	78
Q6Q221	bacOther	89
A4VLP2	bacOther	89
Q9EPF3	animal	83
B2P433	bacA	86
B2P432	bacA	86
B6TR54	plant	83
B9KBT5	bacOther	89
B5ZMX2	bacA	90
Q53M11	plant	98
Q6LZE5	archae	90
B6FG29	bacA	86
A0JWT0	bacB	90
B0VIW3	bacOther	89
C6DGL1	bacA	86
P46516	plant	90
Q9HJU9	archae	84
B4WP44	bacOther	89
A5BXI3	plant	90
Q5S1X5	animal	91
A5E8C1	bacA	90
B0QAF0	bacOther	91
B1JZ09	bacOther	88
A8FWQ2	bacA	87
B2SCM0	bacA	88
Q0F3F6	bacB	89
A9UEZ1	animal	95
B4AW15	bacOther	89
Q2T2U1	bacOther	90
A9NL13	plant	90
O13224	animal	83
C4QAN0	animal	73
B2ILF6	bacA	86
Q4C0I5	bacOther	89
A6L624	bacOther	92
B9ZB33	archae	90
Q81DY2	bacOther	90
A4WLV2	archae	88
C1UL57	bacOther	89
Q6PFS1	animal	95
Q28UK0	bacA	86
Q208N7	plant	96
C0QTQ3	bacOther	89
P40920	fungi	127
A0B581	archae	68
B5HD48	bacOther	89
B2VZY7	fungi	141
B3BDG1	bacA	86
Q0PQ92	bacOther	89
B4QKX4	animal	83
B6B9M0	bacA	86
C5H4M8	plant	109
A7HFM4	bacOther	89
Q5V5N7	archae	79
Q000T2	animal	83
O93591	animal	83
B9SLT1	plant	94
C1YAE8	bacB	90
B2NKC6	bacA	86
B3T5H3	archae	88
B2HF11	bacOther	87
C2MDW8	bacOther	93
B2JTK0	bacOther	89
C2JUC9	bacOther	90
A7JP63	bacB	87
Q8XC04	bacA	86
A7HJ86	bacOther	89
B1J527	bacA	87
B7RN65	bacA	86
Q1C3K9	bacA	86
Q5U130	animal	111
B6K1I9	fungi	89
B6TM52	plant	114
C3VMP2	fungi	151
A0RDP1	bacOther	90
Q72IL2	bacOther	88
C4G9S2	bacOther	91
B4RI57	bacA	86
B1WW89	bacOther	89
Q72IL6	bacOther	85
A9ACP9	bacOther	91
O28308	archae	89
C6T2W1	plant	90
Q5K552	bacOther	89
B2HJW1	bacB	88
C2L005	bacOther	91
Q4SQ32	animal	86
B4SY77	bacA	86
Q2S022	bacOther	89
B8FEZ9	bacOther	89
Q4PLA7	other	90
C0UXW9	bacOther	87
Q13ZT8	bacOther	89
Q5MGL1	animal	83
A2YBC0	plant	96
B1Z465	bacOther	88
B1Z464	bacOther	91
B1Z466	bacOther	89
Q2V9E8	archae	90
Q2RMU6	bacA	86
A1DM46	fungi	139
A4Y7C8	bacA	86
A6L882	bacOther	92
A7NS20	bacB	89
B6U8I5	plant	82
B1BC70	bacOther	90
A7NRF5	bacOther	88
Q27JQ1	fungi	160
Q27JQ0	fungi	86
C1FUQ2	bacOther	88
P02486	animal	83
B4NYS5	animal	91
Q729B1	bacOther	85
Q729B0	bacOther	88
B9PSE4	other	89
A2XEW8	plant	90
A2YAR7	plant	84
A1JT69	bacA	86
Q13QP6	bacOther	92
Q6Q2J1	fungi	138
B4BLY9	bacOther	90
A4ZY18	bacB	88
C2EVW1	bacOther	90
A4I4V9	other	90
Q541V4	plant	86
B1MJW5	bacB	90
C2E3H8	bacOther	90
A4F628	bacOther	91
Q5N1C6	bacOther	89
B2ZHW3	animal	83
A1WAG3	bacOther	90
C6T330	plant	95
B1G457	bacOther	81
B8GYN3	bacA	86
A7ALU6	bacOther	92
Q6W3L1	bacOther	89
B5YFG1	bacOther	89
B1UIX1	bacOther	91
A6X7F7	bacOther	89
B8AWD6	plant	96
Q1HPN2	animal	83
Q1NI80	bacOther	88
A3Q5U9	bacOther	88
C0UVN7	bacOther	89
B4HKP3	animal	100
A6E1D0	bacA	86
B4HKP1	animal	83
A6X7F9	bacOther	89
B7PCG5	animal	109
Q31UU6	bacA	86
Q31UU5	bacA	86
B8C1I4	other	83
Q052T1	bacOther	85
A1ULF0	bacOther	88
C3C1W6	bacOther	90
Q8Y2Y1	bacOther	90
B6YQH1	bacOther	90
A6QJM7	bacOther	86
Q5X395	bacA	87
A9H1P5	bacA	86
Q39930	plant	90
C4AV23	bacOther	90
A5JV80	animal	83
Q545F4	animal	83
A4U381	bacB	89
Q6CYV2	bacA	86
Q7Y099	plant	81
C2BHL7	bacOther	88
A5JV82	animal	93
A6T8V6	bacOther	90
B9S5K5	plant	90
A1T1U5	bacB	90
Q70LH1	bacOther	90
Q3RID5	bacOther	89
A6U5H9	bacA	92
B4PZG1	animal	124
B0NXR6	bacOther	91
B6SLV6	plant	94
A5J5Y3	bacOther	90
Q18F21	archae	89
C3KWZ7	bacOther	88
Q16EM9	animal	121
A9DU04	bacA	86
A5D4M7	bacOther	84
A9NQY5	plant	94
A6YFM2	bacOther	88
Q1JXX2	bacOther	88
Q1JXX1	bacOther	86
A6BWH1	bacA	86
B0KBE9	bacOther	89
B5PR01	bacA	86
Q4J8A6	archae	87
C1NG07	bacA	86
A3YTY0	bacOther	89
C4B4U4	animal	83
Q6KCP0	animal	83
Q6KCP2	animal	83
Q86H60	other	122
Q5EBG6	animal	83
Q2LPQ5	bacOther	89
B6TG53	plant	91
A3Z3G9	bacOther	90
O48865	plant	89
B2SEF1	bacB	87
C2XTK9	bacOther	93
Q312K1	bacOther	87
Q2W081	bacOther	86
A2FKC5	other	87
Q82QP6	bacOther	89
C0QQJ8	bacOther	86
B5ICU4	archae	75
Q5KXZ2	bacOther	91
A9RN10	plant	96
Q8KB28	bacB	89
B5EDM9	bacB	88
Q88U58	bacOther	88
Q6Z2L3	plant	90
Q1M4B8	bacA	85
B3QR17	bacB	89
B9YYM1	bacOther	90
B7FJK9	plant	96
Q1ICW3	bacA	88
B4H1M2	animal	121
B4H1M3	animal	84
B4H1M7	animal	83
B4H1M4	animal	84

B4H1M5	animal	93	P06581	animal	82	A6EPE1	bacOther	92
Q20JZ7	bacOther	89	Q17268	animal	85	B8M4F4	fungi	145
B9LBV0	bacOther	89	B8MTE1	fungi	132	A0FLR8	animal	86
Q01545	plant	92	Q3ISC4	archae	87	Q95P25	animal	83
A3ZTJ5	bacOther	89	B2BXU3	plant	91	Q95P27	animal	105
A8IJR8	bacA	89	A2PL49	bacA	85	B5EAT4	bacOther	89
Q2VNM9	bacA	90	A1SPJ7	bacOther	87	B4FT59	plant	90
A1SUB1	bacOther	73	B3QMB3	bacOther	89	A3TWK2	bacA	85
B7G195	other	83	B7Y7L1	bacOther	91	A2VNG6	bacB	97
A8I6S0	plant	91	B9L593	bacOther	92	A9SWK2	plant	91
Q8IB02	other	95	Q04757	animal	117	B7CWI2	bacOther	90
B1Z8L2	bacOther	87	Q63QV7	bacOther	88	A7FPA7	bacA	86
Q73NX3	bacOther	89	Q63QV6	bacB	91	C1N7X5	plant	95
A9Z5T3	bacA	86	B1Q039	animal	83	Q84J50	plant	90
A9Z5T2	bacA	86	Q97VL9	archae	87	Q94GC7	plant	79
A1RSY5	archae	87	C6T5M6	plant	90	C2NH89	bacOther	90
B9T1D5	plant	89	Q9JK92	animal	142	B6K549	fungi	89
Q663X0	bacA	86	B9HP05	plant	90	C2S3A0	bacOther	90
Q7MQJ3	bacA	85	B9HP04	plant	90	A7PIJ1	plant	90
A0PQK0	bacOther	87	P06904	animal	83	A7PIJ0	plant	90
B8FPS7	bacOther	88	A9LB73	bacOther	88	A7PIJ2	plant	90
C0EYQ0	bacOther	91	A4EEN7	bacA	86	B5QJN6	bacOther	90
C0EYQ1	bacOther	91	C4CK62	bacOther	88	A8HNU7	plant	121
A9URS8	other	89	A6YFL9	bacB	90	A2WLG6	plant	94
B6ZQM7	bacA	86	B0EFK9	other	87	C2GMP7	bacOther	90
C4CJI5	bacOther	92	A3WSV0	bacOther	87	B2FH34	plant	90
B7P6K1	animal	84	Q05VC6	bacOther	103	C3P8X0	bacOther	91
A5FQW0	bacOther	89	A0Y307	bacA	88	C1ZQ54	bacOther	89
B3QZ37	bacOther	89	B5F8A2	bacOther	88	Q6QX37	animal	83
B2GIE8	bacOther	89	B4DNC2	animal	83	A4FFV8	bacOther	90
B4SY76	bacA	86	B8IR16	bacA	87	A9W363	bacA	84
Q05713	animal	83	C0U5K6	bacOther	88	A3NPD9	bacB	90
Q26999	other	95	A9ACQ0	bacOther	88	C1SPN3	bacOther	89
C0PPC9	plant	89	Q99PR8	animal	83	C1SPN2	bacOther	89
Q5UYH0	archae	90	Q2JTG2	bacOther	88	C1KGS9	plant	95
B4FQS7	plant	84	A8LQE3	bacA	86	B4WDT3	bacB	89
B9S1R4	plant	90	C0DAE1	bacOther	91	C3HI28	bacOther	90
Q58FS1	fungi	152	Q1Q416	bacOther	89	C1SJF7	bacOther	88
Q8Z017	bacB	89	Q13XU2	bacB	90	Q03I62	bacOther	90
B0DCN9	fungi	91	Q1Q418	bacOther	88	C0FZW8	bacOther	91
Q9T2L4	plant	95	Q1Q419	bacOther	88	B6VVT1	bacOther	93
Q9T2L5	plant	95	Q8IES0	other	91	Q6USC0	animal	82
B9LTV5	archae	90	B9T5S1	plant	90	Q0FQI6	bacA	86
A9RGS6	plant	90	B9LSK9	archae	87	B7J4U6	bacOther	89
Q2FQN2	archae	90	B9T5S2	plant	90	C4CQ59	bacOther	89
C2Z7N4	bacOther	92	B5XDH1	animal	86	B8B6N0	plant	86
B2S0E9	bacOther	89	B6TIP9	plant	89	Q7RW96	fungi	140
C2CDX4	bacA	85	Q3KF30	bacA	86	Q3JBB5	bacOther	89
B9CGY5	bacOther	88	B3LGB2	fungi	93	B5ISM2	archae	89
B9CGY6	bacOther	91	B8DQK5	bacOther	100	B7JU82	bacOther	90
B8E1J2	bacOther	89	B0BWV0	bacOther	85	B5DGI9	animal	86
B1SGD8	bacOther	88	Q9XDG8	bacB	90	C2SJK2	bacOther	93
A0NRY2	bacA	90	B4FXU3	plant	82	A1RRR8	archae	75
O23640	plant	86	A8M577	bacOther	87	A7FPA8	bacA	86
P41316	animal	83	C4QA74	animal	82	B9T7G2	plant	91
A8KAH6	animal	83	B0UJC9	bacA	87	B9T7G1	plant	90
B8AHC3	plant	97	B2J0U4	bacOther	89	Q329C6	bacA	86
A1EN07	bacA	85	A8LBG0	bacOther	87	A8PLM7	bacOther	83
Q9UBY9	animal	80	B7KD55	bacOther	89	C0P152	fungi	139
A1VBJ7	bacOther	88	Q6LS30	bacA	87	Q0E4A8	plant	90
Q9SSQ8	plant	91	Q2SQW1	bacB	89	Q63HY1	bacB	90
A1VBJ8	bacOther	85	Q21GT6	bacOther	89	P02498	animal	83
A4G7K5	bacOther	90	Q21GT5	bacA	85	Q5P4V2	bacOther	88
O82149	plant	90	B4M9D5	animal	93	Q5P4V3	bacOther	91
B6TLK8	plant	90	Q2SUS8	bacOther	91	A5VN98	bacA	89
Q5NNU7	bacA	88	B3MFI6	animal	128	Q5P4V1	bacOther	89
C3MIQ4	bacA	87	B5AR57	animal	91	B7P7W0	animal	86
C1F1A2	bacOther	89	Q2IRE4	bacA	88	B7RR70	bacOther	88
Q81DZ3	bacOther	92	B4UW89	plant	90	B7RR71	bacOther	89
Q2W374	bacB	88	B5XDZ1	animal	87	B4MN47	animal	97
B6TZJ3	plant	82	A9R4H1	bacA	86	B0KF78	bacA	87
P14602	animal	83	B9R9S7	plant	82	A3MPF4	bacOther	88
Q7U2G3	bacB	97	C1FHI6	plant	95	Q0SAR7	bacOther	87
A5KQQ8	bacOther	91	C2VBC1	bacOther	92	C4K5P1	bacOther	93
A1KF72	bacB	97	B6TVT8	plant	89	A0ZLU0	bacOther	90
Q5NE64	bacB	87	A1JT68	bacA	86	C2TG53	bacOther	91

A5XMJ9	bacOther	90	A3XMR4	bacOther	90	P24623	animal	83
B1JGZ5	bacA	86	Q12UL4	archae	89	Q6YFA5	other	94
C0WMS2	bacOther	90	A4ZF52	plant	90	B2UIT8	bacOther	89
C0WMS3	bacOther	90	A4ZF53	plant	92	B7A9I1	bacOther	89
C1HSW2	bacA	86	A4ZF50	plant	90	A2S3V1	bacOther	88
Q89CQ6	bacA	87	A4ZF51	plant	90	Q03XI9	bacB	90
Q5QUR5	bacA	86	A4ZF55	plant	90	Q148F8	animal	83
A2XTX1	plant	94	B4F9K4	plant	89	Q2S049	bacB	89
B4D446	bacOther	87	B8J4P6	bacOther	89	Q1YZN3	bacA	86
B9EQE2	animal	129	B3LN77	fungi	95	Q2HTU2	plant	90
C0UW20	bacOther	88	Q3JTX8	bacOther	88	Q2Y9V1	bacOther	90
B7PWF5	animal	89	C0H177	bacOther	89	Q57I24	bacA	86
Q1K224	bacOther	87	Q87TQ1	bacA	85	Q57I25	bacA	86
B2VCD6	bacA	104	Q74B23	bacOther	86	C1C116	animal	90
P11890	plant	82	B5HNS8	bacOther	88	B6YWY8	archae	89
Q1PVQ3	bacOther	88	C3IJ02	bacOther	91	B9MYM5	plant	79
A9PA59	plant	90	Q74B24	bacB	89	B5MT67	bacOther	88
Q4JA95	archae	75	B6GWN9	fungi	90	O82545	plant	90
A9TYH4	plant	90	B3NF90	animal	83	Q93TV7	bacOther	87
A6S5D4	fungi	152	A6BYP2	bacOther	89	A1E386	animal	93
Q72C13	bacOther	81	A9P1V8	plant	90	Q7PB94	bacOther	85
C1BNJ4	animal	92	A4FCG1	bacB	85	B7KJ85	bacOther	89
Q738V3	bacOther	90	B4IX99	animal	84	Q67X83	plant	84
Q96331	plant	86	Q9FGM9	plant	84	Q26E01	bacOther	89
A6CVH4	bacA	86	C3G2I0	bacOther	91	A1AS89	bacOther	87
A8ZW92	bacB	89	A6BJL5	bacOther	91	Q39820	plant	90
Q1AXA2	bacOther	89	B4HKP4	animal	111	A1TLP5	bacOther	90
Q2B9P4	bacOther	87	A3B9Q4	plant	84	B3PBC0	bacOther	89
O01263	animal	83	P12810	plant	90	A8P5M5	fungi	117
Q755Y5	fungi	95	P12811	plant	91	Q5ZBP9	plant	86
Q5R9K0	animal	83	C1XMI6	bacOther	88	A1RY97	archae	88
C0R3E6	bacOther	89	B4HKP2	animal	111	C1C2G8	animal	84
B4H1L9	animal	98	Q1ZXL6	other	92	Q0JQV9	plant	90
B4RI75	bacOther	86	A6X7F8	bacOther	88	A4YJK3	bacA	90
Q663W9	bacA	86	A7VF65	bacOther	90	C1C2G0	animal	134
A7QEW6	plant	86	Q8W0Q8	plant	83	B7LK29	bacA	86
B9QG81	other	89	B4HKP0	animal	84	B4G7M3	animal	91
B5EY83	bacA	86	B0QEP4	bacOther	90	A8FR24	bacOther	89
B5IBH1	archae	75	Q86NK3	animal	93	B6A1A9	bacOther	87
B5EY84	bacA	86	A2WJ56	bacB	90	O24082	plant	91
B5BIJ1	bacA	86	Q2RVH4	bacA	88	Q1RI96	bacOther	85
B5BIJ2	bacA	86	C2PEL4	bacOther	92	B9RMP5	plant	104
A1SVR8	bacOther	89	B2BXR1	plant	89	A8ZNQ6	bacOther	89
C1C1T4	animal	84	Q94EN7	plant	94	C2NH77	bacOther	91
C1ST52	bacOther	89	B8FBB4	bacOther	89	B1QNI1	bacOther	88
B7DPN7	bacOther	90	B7L831	bacA	86	A1S769	bacA	86
B9MYJ0	plant	90	A5W6Z1	bacA	87	Q1GJ23	bacA	86
B9BBC0	bacOther	88	Q9UJY1	animal	142	C6TDB4	plant	88
A9NZQ5	plant	90	A6CS43	bacOther	91	A7PII9	plant	90
B5VE37	fungi	95	Q9KFJ9	bacOther	90	A0YF63	bacOther	90
A5EBR2	bacA	88	B4KTG1	animal	93	A7PII4	plant	90
B3HM84	bacA	86	Q8DKI6	bacOther	89	B9M4N9	bacOther	89
B3HM83	bacA	86	Q9ZSY1	plant	90	A8LJM2	bacA	86
A3NTS4	bacOther	90	Q16JF8	animal	83	A9WHJ3	bacOther	89
B4YNR8	animal	91	B1EZ36	bacOther	90	P27880	plant	90
B5GE85	bacB	89	Q16JF6	animal	82	A7PII3	plant	90
Q6I8P6	animal	110	Q16JF7	animal	81	A3W5C4	bacA	86
A3NTS3	bacOther	88	Q16JF4	animal	83	C2AYG7	bacA	86
C1EIT3	plant	129	Q16JF5	animal	83	A6Y8N1	fungi	152
P68283	animal	83	A7X617	bacOther	86	A9E341	bacOther	94
P68280	animal	83	A3KB94	bacA	86	C2AYG8	bacA	86
P68281	animal	83	C1NG08	bacA	86	C3P8Y0	bacOther	90
B4F9E8	plant	99	Q9SBB7	plant	95	Q0IIT6	animal	83
P68287	animal	83	Q9SBB6	plant	92	B4AAN2	bacOther	88
Q092T6	bacOther	88	A5ZU36	bacOther	92	Q5ZSM4	bacOther	89
P68285	animal	83	A5ZU37	bacOther	91	Q8F5V3	bacOther	89
P68288	animal	83	B9J8B9	bacA	92	B0RGR9	bacOther	88
P68289	animal	83	Q8L5F2	plant	90	C3E349	bacOther	92
Q025J8	bacOther	89	A0LUY6	bacOther	87	A1ZSC4	bacOther	90
B6SIH3	plant	90	B5MGV9	bacA	86	A2XEW6	plant	90
C0QIF1	bacB	89	Q8L5F1	plant	90	P29830	plant	91
A6W261	bacA	85	B9JA82	bacA	87	B0CCG0	bacOther	89
C6ZDD3	plant	90	B4QN51	animal	111	B0GBE6	bacA	86
Q05832	plant	90	Q494B4	bacA	87	A2XEW1	plant	90
A4ZF58	plant	90	A5G5D8	bacOther	73	B8HMB6	bacOther	89
C0PD31	plant	89	P24622	animal	83	B1G0A8	bacOther	91

A4TGM7	bacA	86	A4SFB0	bacOther	89	C6T5U3	plant	90
C3KRL9	bacA	85	B4WHN6	bacOther	89	A9RTI7	plant	90
C3KRL8	bacB	87	A9P0F0	plant	90	B8FBC5	bacOther	89
Q8MPA7	other	149	O49710	plant	82	B4L113	animal	84
B2KWT1	bacB	88	A4BPT7	bacOther	93	B4L110	animal	114
B2KWT3	bacB	88	B8FRI1	bacOther	90	B4L111	animal	95
C4K303	bacOther	84	B9MYI7	plant	90	B4L117	animal	115
Q4PM52	animal	86	B5RSD8	bacOther	90	B4L114	animal	84
A9AZ38	bacB	89	B9MYI9	plant	90	B4L115	animal	93
C4K304	bacOther	89	Q6AUW3	plant	96	Q5JEA0	archae	89
B8ADT8	plant	90	B2FH46	plant	90	Q5LC21	bacOther	92
B2IT32	bacOther	89	Q9QZ58	animal	88	A3EJP4	bacA	85
P02488	animal	83	A4KP67	bacOther	87	C6T2F2	plant	90
P02489	animal	83	P04794	plant	90	A8SVW7	bacOther	91
C1XGC3	bacOther	89	P04795	plant	90	A9LB72	bacB	91
P02484	animal	83	P04792	animal	83	Q29F94	animal	84
P02485	animal	83	P04793	plant	90	Q16JG4	animal	87
A5C2G0	plant	90	B6GW11	fungi	108	Q16JG6	animal	83
P02487	animal	83	A4TGM6	bacA	86	C1DIS6	bacOther	89
P02480	animal	83	A1B869	bacA	86	Q9SYV0	plant	90
Q8H1A6	plant	90	Q9A637	bacA	86	C2N0E2	bacOther	90
P02482	animal	83	Q5UZX3	archae	73	Q16JG2	animal	83
P02483	animal	83	Q4EBI7	bacOther	89	C1AJS7	bacB	97
B7RR69	bacOther	89	B6KJY0	other	95	A8RT75	bacOther	91
Q1B3Q0	bacOther	88	A4ZF49	plant	90	Q3ZXT1	bacOther	89
B1FN01	bacOther	88	A4ZF48	plant	90	Q8Z6C0	bacOther	88
A9A7A1	archae	90	A9BGK4	bacOther	89	A7EQW7	fungi	98
B1FN02	bacOther	91	Q9SXP6	plant	92	C2ADR7	bacB	88
B9AEL1	archae	92	A7FS82	bacOther	88	A5TYX7	bacB	97
B3PBM9	bacA	86	Q3B576	bacOther	89	B6T3F5	plant	91
A4JSJ7	bacOther	90	A4ZF47	plant	90	B4V0C4	bacOther	89
A4TXH9	bacA	86	A4ZF46	plant	90	A5B868	plant	91
Q9M566	plant	82	B2FH44	plant	90	Q2JBP6	bacOther	87
A5W385	bacOther	89	Q6HJA2	bacOther	90	B5DFG4	animal	80
C1WN05	bacOther	89	B1KS63	bacOther	88	B9PTP1	other	94
A9SN99	plant	86	B2FH47	plant	86	C2BTV7	bacB	88
A3GYE4	bacA	85	B2FH40	plant	90	A5ETY9	bacOther	89
O44112	animal	83	B2FH41	plant	90	B4PEY1	animal	111
Q9GN07	animal	83	B2FH42	plant	90	Q0B1P0	bacOther	88
Q1V0T5	bacA	90	B2FH43	plant	90	Q8H288	plant	90
Q9DEV0	animal	83	Q24UE1	bacOther	90	B4PEY6	animal	85
C2TG65	bacOther	90	B2FH48	plant	84	B6J9S8	bacA	85
A9HHR8	bacA	86	A3C534	plant	83	B6J9S9	bacOther	87
B0AQP0	bacOther	90	B8I9W4	bacOther	87	Q8TK35	archae	89
Q1AY83	bacB	90	C0B0U8	bacOther	88	Q3I0N4	plant	89
B3AGV6	bacA	86	A4BPT8	bacOther	88	A2XK84	plant	94
C0PP83	plant	94	A8PPU2	bacB	89	B9Q4L3	other	95
B0LSK7	bacOther	90	B8H6W2	bacOther	92	B4PEY4	animal	92
A4VKE5	bacA	87	A9NQ17	plant	82	Q7UF81	bacB	88
A7PIG0	plant	90	Q9C4M2	archae	89	Q7UF80	bacOther	87
B6C1P5	bacOther	89	B9L6U2	bacOther	89	B6UGR5	plant	90
B5YX92	bacA	86	Q67NK6	bacOther	89	Q64T04	bacOther	92
C2QSL5	bacOther	90	B9KS02	bacA	86	A5XUZ4	bacB	91
C1PF38	bacOther	91	B4PEY2	animal	83	A5XUZ3	bacOther	88
B7WXD8	bacOther	89	B4PEY3	animal	84	B0DCP8	fungi	91
Q92SI1	bacA	92	B4PEY0	animal	99	B0GTY1	bacA	86
B9HC69	plant	90	A4VAK4	animal	83	B0DCP5	fungi	91
B4X494	bacOther	89	Q40852	plant	91	A7PJN6	plant	96
A7KCX9	animal	90	A3UZ86	bacA	85	B9SWN0	plant	90
C3X9M1	bacOther	88	O22531	plant	90	A7I106	bacOther	89
A5BUU6	plant	94	Q40851	plant	92	B0DCP0	fungi	91
C0UV66	bacOther	88	B4KWV8	animal	83	A7N1D5	bacA	85
Q7WSY3	bacOther	88	C3X793	bacOther	89	A3DBE9	bacOther	89
Q6NBD9	bacA	87	P12809	bacOther	87	C6T3R3	plant	79
Q5FRB5	bacA	87	B7LK28	bacA	86	B0WT02	animal	92
B0SZR1	bacB	89	B3EKT8	bacOther	89	C3KK66	animal	131
Q1Q6Q3	bacOther	89	B0G2I4	bacOther	92	A4W4S3	bacA	86
Q1Q6Q1	bacOther	88	B5J1Z3	bacA	86	C1XQ89	bacOther	86
A3SV12	bacA	86	B0QEQ3	bacOther	91	A3CVM3	archae	87
B0HJY4	bacA	86	Q1PXW2	bacOther	89	A8GVP1	bacOther	85
A8S842	bacOther	90	A9AEN8	bacOther	85	A5FVK1	bacA	86
Q1IH85	bacOther	89	C0C061	bacOther	91	B6J7I9	bacB	89
Q86KF5	other	149	A9RIP2	plant	94	B9Q9C5	other	95
C2RML2	bacOther	90	C1BTX6	animal	83	A6X7W3	bacA	85
Q9ZSY0	plant	90	Q7A3U9	bacOther	86	A6X7W2	bacOther	87
B6K761	fungi	89	B9GJP5	plant	90	A4T718	bacB	88

Q0JQW1	plant	90	P35385	animal	80	C3XAY2	bacOther	90
Q0JQW0	plant	90	Q6NDQ1	bacA	90	Q0YQR3	bacB	89
A8S8U2	bacOther	65	Q88HP1	bacOther	89	A1TEW5	bacOther	88
B0RYE2	bacOther	89	C3CIC7	bacOther	91	A9F4X2	bacA	86
B4HWF8	animal	91	C0PCL7	plant	80	B4WEI9	bacA	89
B2V9Z8	bacOther	89	B8K8Q2	bacA	85	Q1D8N2	bacOther	73
Q39SC0	bacOther	89	P02474	animal	83	A8PJ71	animal	132
B5CA46	bacA	86	P02476	animal	83	A0LKU4	bacOther	87
P97541	animal	83	Q9AMN8	bacA	87	A8WCV1	plant	90
Q1QXM5	bacB	89	A3GL01	bacA	85	A8WCV0	plant	90
B8HF31	bacOther	88	P02472	animal	83	A8WCV3	plant	90
Q8MJ36	animal	142	Q6FVM1	fungi	93	A8WCV2	plant	90
B6BG55	bacOther	91	P02479	animal	83	A8WCV5	plant	90
C1UBM4	bacOther	91	C0ZG78	bacOther	88	A8WCV4	plant	86
Q550E9	other	156	Q629T3	bacB	90	A0K0K3	bacOther	88
B9HQU3	plant	90	B9GUS2	plant	93	B2FH53	plant	89
Q1QF72	bacOther	86	B2IBF6	bacA	90	B2FH52	plant	87
A0LSH2	bacB	88	P42929	animal	83	B0JSS2	bacOther	84
Q8PBS2	bacOther	89	A2BM79	archae	73	B2FH50	plant	85
A4U847	animal	83	Q738W4	bacOther	91	B2FH57	plant	89
P30236	plant	90	B4X4U5	bacA	87	B2FH56	plant	89
Q162V2	bacA	86	B4T3T8	bacOther	88	B2FH55	plant	89
Q0YPZ2	bacOther	89	B4L112	animal	90	B2FH54	plant	77
A7PIH7	plant	90	C4FCZ7	bacOther	91	Q16JG1	animal	83
Q23951	animal	132	B2PHX8	bacA	86	B2FH59	plant	100
A6N9U9	animal	83	C1PB33	bacB	90	C6TM72	plant	88
Q6P3C8	animal	83	Q7D321	bacA	85	P31170	plant	85
Q2CB60	bacA	86	A9IVE6	bacA	88	A4G7H8	bacOther	90
Q29Q59	plant	90	Q9ZVC6	plant	79	Q73TL7	bacB	90
A6C3F7	bacOther	88	B8A1I4	plant	92	Q16JG3	animal	83
Q6F862	bacA	88	Q98GS9	bacA	90	B6C2B6	bacOther	89
Q9EPX0	animal	142	P57640	bacOther	88	A4S909	plant	93
B6TVC4	plant	81	C0ZSY0	bacOther	88	Q74B03	bacOther	90
B7PIN1	animal	83	Q9VL41	animal	91	A3FPF6	plant	87
B8DIS7	bacOther	123	A2BHS1	animal	95	Q40847	plant	82
C6TNH7	plant	95	B1C5G9	bacOther	91	Q01544	plant	91
Q8QFU5	animal	83	B9I8F5	plant	90	Q24R86	bacOther	88
A4JD75	bacOther	88	B2NKC7	bacA	86	Q5MCR6	plant	90
Q1E6R4	fungi	107	B4RI58	bacOther	87	A2Y842	plant	80
B8FM33	bacOther	89	Q2NBM1	bacOther	87	A3ESL3	bacOther	88
A8CNQ3	bacOther	89	Q7EZ56	plant	79	C1TDY3	bacOther	76
A8KUB4	bacB	91	Q7EZ57	plant	86	Q6ZWL5	plant	85
Q6CYV3	bacA	86	C0YW51	bacOther	90	A0RYP0	archae	73
B0M9N7	bacOther	91	B8HUD8	bacOther	89	B5UU81	bacOther	92
Q8YW07	bacOther	89	B4RI50	bacA	86	C4DBR8	bacOther	89
C3AL76	bacOther	92	A5C1L3	plant	90	A3QEV2	bacA	86
A8HT80	bacA	88	A7C679	bacOther	89	C0UVF9	bacOther	88
C0YDJ9	bacOther	90	B9HC70	plant	90	B3Q6N4	bacOther	87
Q891B1	bacOther	88	C6K8Q9	other	93	B2K7E5	bacA	86
B6TMR8	plant	86	B9M1W9	bacOther	89	Q5HDG8	bacOther	86
A5A8V1	plant	95	A8Q0M9	animal	83	C3KLU7	bacOther	89
O14368	fungi	91	C0UV77	bacOther	89	Q2NQ17	bacOther	92
A1AKS8	bacOther	86	B6EMG8	bacA	86	C4FC38	bacB	88
A2I3W3	animal	83	Q9ZSX9	plant	90	Q30WR1	bacOther	88
C2MKD5	bacOther	90	Q9ZSX8	plant	90	A4WYL7	bacOther	89
B1T781	bacOther	88	B3M6F2	animal	129	Q92IQ7	bacOther	85
B9HTE1	plant	95	Q93141	animal	83	Q60A86	bacOther	89
A7RMQ6	animal	82	Q72I75	bacOther	89	A9ILR8	bacA	89
C0YBJ9	bacOther	90	Q72L60	bacOther	85	A3PVI8	bacB	88
C2YA58	bacOther	92	B6SIX0	plant	90	B1MET7	bacOther	87
Q6L128	archae	86	A9P2I5	plant	90	B6TGQ2	plant	90
B7RR56	bacOther	93	B9RE65	plant	70	O81961	plant	86
Q30WR0	bacOther	85	B6U2K9	plant	97	B5FMC8	bacA	86
Q0RPJ9	bacOther	90	Q03B95	bacOther	90	A7B048	bacOther	91
B5RB54	bacOther	88	Q0CS21	fungi	120	Q1GDN6	bacA	86
Q1PCB4	animal	93	A4CXA6	bacOther	96	C3PVB6	bacOther	96
P02493	animal	83	C4ZYW3	bacA	86	Q62FI3	bacOther	90
P02492	animal	83	B1ZAF3	bacOther	89	A3WSU6	bacA	87
B5Q695	bacA	86	A9RGT5	plant	90	C3IYY7	bacOther	91
B5Y7P2	bacOther	89	C4ZYW4	bacA	86	Q3JAU4	bacOther	89
P02497	animal	83	Q98DG1	bacOther	89	B7H509	bacOther	92
P02494	animal	83	Q98DG0	bacOther	88	Q2CFL0	bacA	86
Q1PCB6	animal	93	P46730	bacOther	87	B1FD37	bacOther	89
Q3M1B2	bacOther	89	P46731	bacOther	87	Q04CB2	bacOther	90
Q3M8K1	bacOther	89	A8DJJ8	bacOther	89	B9DXQ1	bacOther	88
C3X318	bacOther	91	B2XVV2	other	95	C1ZKW4	bacOther	85

C2QBK5	bacOther	92
A9P171	plant	90
C1SQ62	bacOther	89
Q2K4A9	bacA	87
A5D0B6	bacOther	89
B8KKM4	bacA	86
B1EIJ8	bacA	86
B0N202	bacOther	91
Q57733	archae	90
Q8L5D5	plant	91
Q8L5D6	plant	91
Q8L5D7	plant	82
B3EA90	bacOther	90
A3YXD6	bacOther	94
B9HZ23	plant	80
C3Q513	bacOther	93
B4X941	plant	90
Q3IZ35	bacA	86
C4DD67	bacOther	88
B4G250	plant	90
A4RN64	fungi	115
Q46QT6	bacOther	91
B3NPQ4	animal	128
Q7U3R4	bacOther	87
A5BIM9	plant	94
Q38ZM7	bacOther	90
Q3LGX1	animal	84
A3IYE9	bacOther	89
Q3LGX2	animal	84
A1RXI1	archae	91
A7ATV2	other	95
A1B6T2	bacA	85
A1B6T3	bacOther	87
Q1QP62	bacA	89
B4SEH4	bacOther	89
A8VQC8	bacB	90
A9KAK1	bacOther	88
A7QPH7	plant	82
A9KAK2	bacOther	90
Q9ZFD1	bacOther	89
B4S543	bacB	89
O24247	plant	91
B8IH28	bacOther	91
B1TBX9	bacOther	91
B9MCW3	bacOther	90
B4U8G1	bacOther	89
Q1YFC4	bacA	93
Q74LK4	bacOther	90
B8LRE5	plant	86
Q1GRE0	bacA	86
B4FES7	plant	85
A6ZL16	fungi	95
B0Y0M6	fungi	92
C0FC53	bacOther	91
Q11C44	bacA	88
B8H6B0	bacA	90
A7IGY3	bacA	90
C2YJI7	bacOther	90
C0XJ10	bacOther	90
C0XJ11	bacOther	90
Q6DQI8	other	89
A6P0V3	bacOther	90
A7UDX2	animal	93
B4HIV7	animal	83
A8WJJ4	animal	83
B1IGL6	bacOther	88
C1C2K8	animal	84
Q5PR64	animal	83
Q0B1P1	bacOther	91
Q1Q6Q2	bacOther	88
A3WSG7	bacA	90
B9I303	plant	79
Q5X234	bacOther	89
P30221	plant	90
Q7MRJ3	bacOther	89
P30222	plant	86
A7HEY0	bacOther	89
C1GQS6	fungi	131
B2TUT6	bacA	86
B2TUT5	bacA	86
B3RCY5	bacOther	91
P31673	plant	90
B3EA88	bacB	89
B4DL87	animal	83
C3LJK5	bacOther	90
A3SR97	bacA	86
Q0SYN0	bacA	86
A0KEA7	bacA	85
A0KEA5	bacA	85
B5CTK5	bacOther	91
B0U3C8	bacOther	89
B4AZ68	bacOther	89
Q13ZT9	bacOther	88
Q60GF8	animal	86
A3NDH8	bacOther	88
A3NDH9	bacB	91
Q8L7T2	plant	87
C0YBK0	bacOther	88
A3WYD2	bacB	89
Q21CA2	bacA	90
Q00TK2	plant	93
Q5R1P6	animal	83
P30219	animal	104
B9XSI4	bacOther	89
Q5R1P5	animal	90
A8T622	bacA	85
O14558	animal	83
B6ZQM6	bacA	86
A5TM99	bacOther	90
A7HUK2	bacA	85
B8FAU4	bacB	89
B8NEB7	fungi	111
A9QVI5	plant	90
B5XFE4	animal	86
Q7XE46	plant	83
Q0RPI6	bacOther	87
B1JGZ6	bacA	86
A9TAL4	plant	90
B0UP30	bacA	84
Q5WUQ2	bacA	87
A3SA49	bacA	86
A4YRI7	bacA	85
B6UD04	plant	91
Q40056	plant	91
Q40057	plant	86
B1QRU0	bacOther	88
Q2SF12	bacOther	89
A0NNK7	bacA	86
Q7WMZ1	bacOther	87
Q65HW1	bacOther	90
B8ANK2	plant	81
C4LVW9	other	84
Q6L4S2	plant	80
C0CJM8	bacB	90
P34696	animal	82
C4KKC9	archae	75
C2EAL7	bacOther	90
Q19228	animal	82
B5RRP9	bacOther	89
A6FPL4	bacB	96
O67316	bacOther	89
C0FPB4	bacOther	92
Q8PXG3	archae	73
A5AND9	plant	90
C4FKB7	bacOther	89
C2K3X8	bacOther	86
B5PK58	bacOther	88
A7PIE2	plant	90
C1SKB1	bacOther	89
Q0AZD7	bacOther	88
A3FMM9	animal	83
A9NMS0	plant	92
C4I0D6	bacA	86
Q9RTR5	bacOther	84
Q01J40	plant	94
B5AR56	animal	91
Q46QT5	bacOther	88
Q5SI90	bacOther	88
Q0BSE6	bacA	86
Q6ACF4	bacB	88
Q8ZTS4	archae	75
Q5SI94	bacOther	85
B0DXV1	fungi	91
Q2FEB1	bacOther	86
Q6R2J2	animal	83
B7J6T3	bacOther	87
B3LBK6	other	91
Q4MTS3	bacOther	92
C4FKC7	bacOther	86
Q4N318	other	95
C6SXG1	plant	90
B9IDI9	plant	76
A2RZM8	bacB	90
C6SWI1	plant	90
C6SWI2	plant	72
B0R313	archae	90
A6D7Y5	bacA	85
A3SSJ9	bacA	86
B0R2M3	archae	73
A0KXI1	bacA	86
A1WIF6	bacOther	74
Q10RJ2	plant	83
C3A5F3	bacOther	93
B7MGA8	bacA	86
Q53595	bacOther	89
A9YME5	animal	94
Q0B1N9	bacOther	89
Q29NG9	animal	91
B8LQJ4	plant	90
B3MPL0	animal	91
Q06823	bacOther	89
B3BDG2	bacA	86
B7N1Z1	bacA	86
C1DXW7	bacOther	86
Q0V0I8	fungi	97
C4IU58	bacA	88
B4IWP6	animal	83
C0PPG1	plant	97
Q0FEY9	bacA	86
B4LGT1	animal	84
Q9KVX0	bacA	85
B4QMS7	animal	92
B9P9K4	plant	90
C6TC68	plant	80
A4J0D8	bacB	89
B5VG66	fungi	93
B5YIQ1	bacB	89
Q2B3Z2	bacOther	91
A9NNU0	plant	82
Q09648	animal	83
B7YDF0	bacOther	88
B4QMS8	animal	84
A9NS53	plant	91
A9T060	plant	90
Q64CD5	archae	89
B5X9K7	animal	87
B9GIX7	plant	94
A8NZ97	animal	83
B4J4G2	animal	93
A6R3A3	fungi	139
B4HAQ0	animal	125
Q1AMF4	animal	104
A5GWM8	bacOther	89
Q0DY72	plant	97
C3JPP0	bacOther	88
Q8ZPY6	bacOther	88
A3EUL0	bacOther	90
B5XDG3	animal	87

B9GH40	plant	89	B9EQI5	animal	101	B6EXX3	plant	90
A3LD86	bacA	87	B6ARI9	bacOther	88	A8XKR5	animal	133
Q2PPK1	animal	83	B2K7E6	bacA	86	C6T4P8	plant	79
Q8THE3	archae	73	B1ZWT7	bacOther	83	A8N559	fungi	91
B7EZJ7	plant	90	B2GDH3	bacOther	90	A0LM85	bacOther	89
A1VFV2	bacOther	88	P96193	bacA	87	C2FPH9	bacOther	90
A1VFV3	bacOther	85	P13853	plant	90	Q5R1P4	animal	83
B9ZDK4	archae	92	C2AJ40	bacOther	88	B2G3H0	bacOther	88
A9GVB2	bacOther	84	Q1GRD9	bacB	87	C1XFC0	bacOther	85
A9GVB5	bacOther	88	Q329C7	bacA	86	Q8MPA5	other	155
B1GII5	bacOther	91	B4RI37	bacOther	89	C0LAM0	fungi	91
B9Y451	bacOther	91	Q5Z1K6	bacOther	88	A8ZX67	bacOther	89
B3GS75	plant	90	C3X796	bacOther	87	A8ZX66	bacOther	88
B9Y452	bacOther	91	Q86G69	animal	84	C2HBE5	bacB	89
B3A2D6	bacA	86	A5AG87	plant	90	Q40510	plant	90
B4I8R4	animal	93	C6SYL5	plant	91	A4AVG1	bacOther	89
B3A2D5	bacA	86	Q13ZX1	bacOther	88	A9FXE9	bacA	86
B6H9F1	fungi	97	B6INQ2	bacA	86	A6QY49	fungi	101
Q6MPD8	bacOther	89	C0BD51	bacOther	92	C6SVX0	plant	90
A4ESM0	bacA	86	P29778	animal	85	B4P9R1	animal	128
B9XGQ1	bacOther	89	A2ZQ23	plant	78	C1N5X6	plant	126
B2IWQ0	bacOther	89	P09886	plant	86	A9CX83	bacA	92
Q8G6R2	bacOther	107	B5PBK2	bacA	86	B4LGT0	animal	84
Q9X9N3	bacOther	90	A5N431	bacOther	88	A6BE80	bacOther	91
C1H3L3	fungi	100	B5PBK1	bacA	86	B6IZV4	bacB	89
A7V2Z3	bacOther	97	B5QDT2	bacOther	88	B4LGT4	animal	90
A1HQE7	bacOther	88	B4IYH1	animal	115	A5JV84	animal	83
Q6GL34	animal	95	A8WY16	animal	82	Q97AM1	archae	84
C1PT25	bacOther	90	A3V5H2	bacA	86	A5U456	bacB	88
B2N6C4	bacA	86	A8WY17	animal	82	B3PZW3	bacA	87
A7HIY1	bacB	89	C1K660	animal	83	B4EVJ7	bacOther	88
Q87C16	bacOther	89	A5ULJ7	archae	92	B1TBX8	bacOther	88
B4D447	bacOther	89	P30218	animal	106	C1QD83	bacOther	88
B7H519	bacOther	90	C3NCK1	archae	87	B6Y8Z4	bacOther	89
B1Z4F8	bacOther	90	A8YDU2	bacOther	89	Q46E59	archae	89
C2UDC0	bacOther	92	B6AKR0	bacOther	90	B6H5V7	fungi	144
Q5PKS6	bacA	86	A5A8V0	plant	92	A3V9Z9	bacA	86
Q5PKS5	bacA	86	A5A8V3	plant	92	Q8L8S1	plant	90
A2S5H0	bacB	91	A5A8V2	plant	92	Q6G6T4	bacOther	86
Q20165	animal	83	C3VMY8	animal	83	B1WWB7	bacOther	89
B0R9P1	archae	79	A5A8V4	plant	92	C2NYA5	bacOther	92
B4UY27	bacOther	89	A4BE31	bacA	86	B3ZUN3	bacOther	91
Q4C3P7	bacOther	89	A6Q779	bacOther	89	B5PR02	bacA	86
A2XCQ1	plant	86	B6G7P4	bacOther	88	B4TMX7	bacA	86
C3J7G8	bacOther	90	B8LM88	plant	85	Q033V8	bacOther	90
B4IYY3	animal	97	Q4UJB0	bacOther	85	B3NVQ2	animal	124
A6F829	bacA	89	Q4UJB1	bacOther	89	A3U7U0	bacOther	91
A6F828	bacA	86	A9NFB2	bacOther	90	Q14FL7	bacB	87
B3EA89	bacOther	86	Q552K9	other	146	B3MQA4	animal	125
C2RMK2	bacOther	92	C1WNQ7	bacOther	87	B1Z7M4	bacOther	87
Q0C682	bacA	88	A1AW82	bacOther	87	B1Z7M3	bacA	86
Q9FHQ3	plant	95	Q2WG64	animal	83	Q08275	plant	89
Q6J0T4	plant	90	A6E6F1	bacOther	89	Q8LCB4	plant	84
C1TPD4	bacOther	88	B9LSA4	archae	125	P06582	animal	82
C1Y0W3	bacOther	88	C0UNJ7	bacOther	88	A1V2X8	bacOther	88
Q2KYH5	bacOther	87	B9IZR3	bacOther	90	A1V2X9	bacOther	90
A2EJL4	other	87	B9LSA3	archae	89	C4DDZ7	bacB	86
C2VTA7	bacOther	90	A1AKS9	bacB	89	C4K151	bacOther	85
A8XV09	animal	82	A4ZX74	plant	89	B3PZA6	bacA	90
B9I224	plant	86	A7VBU8	bacOther	92	C1BP33	animal	79
A2S3V0	bacOther	90	P30693	plant	90	Q9ZSZ0	plant	90
B0R6E3	archae	91	C0Q740	bacOther	88	Q1PXG9	bacOther	89
B2UIS6	bacOther	90	Q2YIE9	bacA	88	Q3AUK8	bacOther	89
Q91308	animal	87	B6A4X2	bacA	85	B8BN43	plant	83
B8A113	plant	91	B2V6N1	bacOther	86	B9F2C0	plant	100
Q30ZU1	bacOther	81	B9XRT8	bacOther	87	B9R8U2	plant	82
Q0AFP2	bacOther	89	B9XRT7	bacOther	89	B6T5G0	plant	84
Q1ARJ6	bacB	89	B3AXF7	bacA	86	O48898	plant	89
B9L1K2	bacOther	87	Q6Z7V2	plant	84	A8TI16	archae	90
B5BZ61	bacOther	88	Q57A32	bacA	88	B6WUU3	bacOther	100
Q9ZP84	plant	90	P0C060	bacA	86	B4RHY8	bacOther	91
A9N391	bacOther	88	C0YR18	bacOther	87	Q8VXJ3	plant	90
C0GND0	bacOther	88	C2FYG4	bacOther	89	Q8VXJ4	plant	90
C0GND1	bacB	85	B5TGQ8	animal	83	Q551F8	other	174
A3WC19	bacA	87	A9QVH3	plant	86	C3L2B5	bacOther	88
A3K904	bacA	86	Q9M670	plant	80	C4APC3	bacOther	88

C4APC2	bacOther	90
Q9RVB5	bacOther	88
Q63VB9	bacOther	90
B9IA42	plant	98
B6QVV3	fungi	131
A5XRW8	bacOther	88
A5XRW7	bacOther	90
B1M1B4	bacA	86
B2ZBG8	plant	90
Q5T5Q2	animal	80
A3MWN1	archae	88
A3PN62	bacA	86
Q2BEX9	bacOther	87
Q12E92	bacB	91
A8WY15	animal	82
Q8LBH7	plant	90
Q8LD91	plant	96
B0NH96	bacB	91
B0M1A7	plant	95
B6B8E6	bacOther	96
Q83CE9	bacB	89
A8G7R9	bacA	86
A4WLE3	archae	75
A6LM31	bacOther	89
Q0BJX2	bacOther	87
A8RTQ4	bacOther	92
B8I8D2	bacB	88
Q3APD3	bacOther	89
Q8G2Z4	bacA	89
A8RTQ3	bacOther	91
A5J5Y4	bacOther	88
O69241	bacA	85
O69242	bacA	85
O69243	bacB	86
A9XBI3	plant	90
Q8PZK9	archae	89
B6U175	plant	99
A1ATK1	bacOther	89
C6T1Y1	plant	90
A5FUZ6	bacB	89
A7SQF4	animal	97
A7SQF6	animal	83
A6C0C3	bacOther	89
Q40867	plant	90
Q40866	plant	90
Q40865	plant	90
C4RKY5	bacOther	87
B1FU61	bacOther	89
B1FU62	bacOther	88
B1FU63	bacB	92
B4H2A3	animal	83
A4JD76	bacOther	91
B0T680	bacA	89
B1GIH5	bacOther	90
Q8TUD5	archae	89
B1Q6U9	bacOther	88
A1BET9	bacOther	89
B4EEH1	bacOther	90
C0RK03	bacA	88
C4KV48	bacB	91
C0PSL0	plant	86
Q6RFM0	plant	90
A9ZZB9	bacA	86
A9NK63	plant	91
Q8ZTN4	archae	75
B4BRE7	bacOther	88
Q04H04	bacOther	90
C6SV69	plant	92
A7HPH8	bacA	88
Q109M7	plant	84
A2VJD9	bacB	88
B2AM23	fungi	156
B1EIJ7	bacA	86
B1IYQ7	bacA	86
Q6DUA8	other	95
A6WCM8	bacOther	87
B9L020	bacOther	89
B1IYQ8	bacA	86
B7ZRQ5	animal	83
C1VAD6	archae	79
Q1NXJ6	bacOther	89
Q81R10	bacOther	91
A6CAR4	bacOther	86
A2S5G9	bacOther	88
B5ZQ18	bacA	87
A0B7C0	archae	72
Q8PX03	archae	89
A5IK41	bacOther	89
A2X246	plant	90
B4WSZ5	bacOther	89
B4S6P8	bacOther	89
Q3A7M6	bacOther	86
C3WB02	bacOther	91
Q91311	animal	83
A4WP08	bacA	86
O64564	plant	72
Q0W6B5	archae	73
B8E9E2	bacA	86
Q4WNG3	fungi	139
C1APV1	bacB	88
C2F1P3	bacOther	90
C0GSG2	bacOther	89
B5ERR0	bacOther	89
Q084A0	bacA	86
Q9VSA9	animal	83
Q3XX56	bacB	89
Q6G1G0	bacA	89
Q752Z6	fungi	95
B5NDS5	bacA	86
A9PFJ9	plant	98
A7F5L9	fungi	148
C2A5F4	bacB	88
Q72EC0	bacOther	88
Q72EC1	bacOther	85
B1HHR6	bacOther	88
C4RBK2	bacOther	88
Q41815	plant	94
Q10FT7	plant	79
B0HJY3	bacA	86
Q9LPG9	plant	90
A4YWX0	bacB	89
B6T1R8	plant	85
C0RGL7	bacA	89
B5G209	animal	88
Q5EAC9	animal	142
C1YR00	bacOther	87
Q9X9C3	bacOther	90
Q1N8K9	bacA	105
Q3ITA4	archae	96
Q6CD63	fungi	93
B5IM58	bacOther	100
A6Q780	bacOther	90
Q67QW2	bacOther	86
A5A8U5	plant	86
A5A8U6	plant	95
A5A8U7	plant	95
A5A8U0	plant	85
A5A8U1	plant	85
B3XNA0	bacOther	90
A5A8U3	plant	87
B6C4X3	bacOther	89
A5A8U8	plant	95
A5A8U9	plant	92
B6C4X4	bacOther	89
Q2WFK9	plant	90
B1X3A5	bacOther	89
A9WX80	bacA	88
Q5SKS2	bacOther	85
C3I0B9	bacOther	90
A9TWQ2	plant	90
C2CYT7	bacOther	90
B8GJM7	archae	92
A7NYP2	plant	90
Q2KAT7	bacA	85
Q2KAT6	bacOther	87
B9MJU9	bacB	88
A7RMQ7	animal	97
Q6DG35	animal	83
B9L2N0	bacOther	92
A9VTY2	bacOther	93
Q2NX42	bacA	103
A0LJ27	bacB	89
Q2RL87	bacOther	91
Q7XZ71	other	117
A7UZZ9	bacOther	92
A1AHM2	bacA	86
B1M1B3	bacOther	87
B7FK78	plant	79
Q3SW62	bacA	90
Q16CG4	bacA	86
Q9VWG1	animal	89
A8EIA6	bacOther	88
A8EIA7	bacB	91
P94898	bacOther	90
P02499	animal	83
B0NKH9	bacOther	91
B7QVQ7	bacA	86
Q9V1L0	archae	89
A4SH53	bacA	85
B3E7K3	bacOther	89
A8G7R8	bacA	86
C1XQN9	bacOther	88
B6QRH6	fungi	157
A3Q9M0	bacOther	89
B5TGR2	animal	83
B1KXD4	bacOther	88
P0C054	bacA	86
P0C055	bacA	86
Q2JL65	bacOther	89
Q28ZF4	animal	128
B8D8A0	bacOther	88
A7MPZ0	bacA	86
C3BK20	bacOther	92
Q1GP87	bacA	88
A1B453	bacA	86
C0Y6K2	bacB	91
Q4WV00	fungi	92
A0LC76	bacOther	89
A7NEZ3	bacOther	87
A8N561	fungi	91
C3MKA3	archae	75
Q5L3D9	bacOther	88
Q84Q72	plant	90
Q84Q77	plant	90
Q89C37	bacOther	89
B1X9B6	bacA	86
B1X9B7	bacA	86
A8ZX51	bacOther	89
Q2NH97	archae	90
Q0G5S4	bacA	92
A9VY78	bacA	86
C4C7M6	bacOther	89
Q943E6	plant	90
Q943E7	plant	90
Q943E9	plant	91
Q03928	bacOther	90
B9CMA0	bacOther	88
Q7WBG9	bacOther	87
Q2T6S8	bacOther	89
A2FKF2	other	87
A3XZZ5	bacA	85
Q29IV3	animal	125
B9NMH0	bacA	86
B6H317	fungi	106
C2KTB0	bacB	88
Q74MA9	archae	89
P02518	animal	111
P02519	plant	90

P02516	animal	99	B0NQA6	bacOther	92	C1PB40	bacB	90
P02517	animal	83	Q95661	plant	87	C2G8F2	bacOther	86
C2NYB6	bacOther	90	P02478	animal	83	C1GHG3	fungi	100
P02515	animal	92	Q4URT0	bacOther	89	Q57Q43	bacOther	88
P02512	animal	83	Q0TB20	bacA	86	A5AT75	plant	90
P02513	animal	82	C0Q251	bacA	86	B7M4H5	bacA	86
P02510	animal	83	C0Q250	bacA	86	B7M4H6	bacA	86
P02511	animal	83	Q9ZPQ0	plant	72	C2HM07	bacOther	90
Q0HVN5	bacA	86	A0QZ83	bacB	88	C2W7P3	bacOther	92
A1TC18	bacB	88	B3M6F1	animal	139	A7QIP4	plant	89
C1ZXT9	bacOther	89	Q30VU1	bacOther	89	C0PLX5	plant	90
A9NU79	plant	91	A7M0U1	bacOther	92	A4WL81	archae	75
A1V0E1	bacB	91	B9FK35	plant	96	B2PHX9	bacA	86
A1V0E0	bacOther	88	Q9LS37	plant	92	A3UH27	bacA	88
B9JS60	bacA	88	A7SQF7	animal	97	C2F8E9	bacOther	90
B5QU36	bacA	86	C6T1V2	plant	90	Q9LNW0	plant	90
B5QU37	bacA	86	B0UN18	bacOther	89	B5WIC4	bacB	90
Q18DP6	archae	79	Q5MCP0	animal	83	B4EEH0	bacOther	88
B0S9H4	bacOther	88	B9MYM7	plant	79	A6W4Q7	bacOther	87
B0S9H5	bacOther	89	A7GPB1	bacOther	90	Q01QQ8	bacOther	89
Q46BJ5	archae	89	P0C057	bacA	86	B5XDZ3	animal	87
Q220H0	bacOther	89	Q7YWK6	other	95	P19244	plant	90
Q63BT2	bacOther	90	B2J3W2	bacOther	89	P19242	plant	90
A9XBI2	plant	90	Q566R8	animal	83	P19243	plant	90
Q3SND9	bacA	89	C1TAI7	bacOther	89	Q27SL3	bacOther	90
A0PX96	bacOther	90	B9BBB9	bacOther	91	Q43660	plant	88
B9PKA9	other	95	B5J8W9	bacA	86	C6THQ1	plant	92
A6MYV3	bacOther	85	Q3A9H1	bacOther	90	Q64DP6	archae	89
A6MYV2	bacOther	89	C1F1Y8	bacOther	90	Q04TQ0	bacOther	85
A3MX60	archae	75	B0GBE7	bacA	86	Q04TQ1	bacOther	87
Q7M2W6	animal	83	C3D180	bacOther	91	B9E7L3	bacB	90
B9BAG5	bacOther	85	Q2KHU9	animal	88	A2I490	animal	85
C3QRG3	bacOther	92	B0GTY2	bacA	86	B5Y4C3	other	95
Q9HZ98	bacA	87	P19752	fungi	158	B3ZUP5	bacOther	90
B3WW25	bacA	86	B4L811	animal	125	A1D5W7	fungi	126
B3WW24	bacA	86	Q8AAA0	bacOther	92	Q31E11	bacOther	89
Q1MMC7	bacA	90	Q05557	animal	83	Q7Y0A2	plant	94
A6FSL9	bacOther	89	A5D2M5	bacOther	90	B8GB01	bacOther	89
A3HWK2	bacOther	89	C3MTC4	archae	87	B1L3V3	archae	77
C4J5C1	plant	84	O28973	archae	73	Q8L470	plant	87
Q2IP34	bacOther	89	B6R2H6	bacA	90	Q2IWA6	bacOther	87
A7UNS9	plant	91	B6SJE9	plant	89	B5NUL2	bacA	86
B2GWJ2	bacOther	90	Q2SUS7	bacOther	88	B5NUL3	bacA	86
B2GWJ3	bacOther	88	A0RXR5	archae	87	B7KXM2	bacB	89
C0P8J4	plant	90	Q0UTQ3	fungi	236	A5A8T7	plant	89
A3DK41	bacOther	88	C2LWP1	bacOther	86	C1DVH9	bacOther	89
A7P4Q3	plant	70	A5V3X1	bacA	88	A5A8T4	plant	89
B2IXS3	bacOther	89	P02506	animal	83	C0PSL4	plant	86
B5EDN0	bacOther	86	Q6DJI2	animal	83	A5A8T9	plant	85
B7VBP4	bacA	87	C0G1Y9	archae	89	A5A8T8	plant	89
P02477	animal	83	C4ELG3	bacOther	88	A7VJB3	bacB	88
Q8N241	animal	80	A4IFQ9	animal	83	A1RRY3	archae	75
B5N0T0	bacA	86	B5W5D6	bacOther	89	B3M964	animal	83
Q63VC0	bacOther	88	A9AE77	bacOther	58	B9HQH9	plant	79
A6FV78	bacOther	86	B5EKD0	bacOther	89	Q89WG1	bacA	90
P02470	animal	83	Q546L9	animal	83	B7GFT1	bacOther	87
B6BS30	bacA	90	B5P7T6	bacOther	88	C3I0A6	bacOther	92
B3GK93	animal	106	P82531	animal	83	C1YJ13	bacOther	88
B9RXN2	plant	62	P82533	animal	83	Q9SMF2	plant	90
A1USG7	bacA	88	Q5V8K5	fungi	91	Q1C3L0	bacA	86
A3PG77	bacA	86	Q0BGM3	bacOther	91	B9XRD9	bacOther	87
Q9QZ57	animal	90	A8NJG2	animal	83	B9XRD8	bacB	89
B5G213	animal	88	A5AFX4	plant	82	B9RQT7	plant	90
B4UZC5	bacOther	89	A8YX44	bacOther	90	C6TJG3	plant	80
B3IKH2	bacA	86	Q0BGM4	bacOther	88	A3IKW0	bacOther	89
A8SC82	bacOther	90	A8F352	bacOther	89	A6A4U2	bacA	85
A6N6X0	animal	83	B9DLB1	bacOther	90	A8PSZ9	animal	83
A3X4W6	bacA	86	C1BXD1	animal	86	A6XXH0	bacA	85
Q5E8Y8	bacA	86	A5I023	bacOther	88	B9RQT8	plant	91
A6BE79	bacOther	92	A7RA96	bacOther	89	B9RQT9	plant	93
B7JMH7	bacOther	91	B7UMF3	bacA	86	C1G9M7	fungi	131
P68286	animal	83	A1QZN2	bacOther	89	C3KMZ6	bacA	85
Q8GWH1	plant	91	C1FNQ3	bacOther	88	Q00445	plant	92
C1D2D1	bacOther	88	B4R8Z7	bacA	86	Q5XLC5	other	95
A8EY26	bacOther	85	A2Z584	plant	100	B3YEC8	bacA	86
Q8PNC2	bacOther	89	B2TFQ6	bacOther	88	B2HN40	bacOther	93

B8E2I9	bacOther	89	Q1PCB5	animal	83	P27397	plant	90
C0P4A5	plant	79	B1T782	bacOther	89	P27396	plant	90
Q8NJ78	fungi	91	Q1PCB7	animal	83	A3AGD3	plant	90
B6VU66	bacOther	96	C0PS07	plant	86	Q91312	animal	83
Q27354	other	95	C4K5Z9	bacOther	93	C1VB95	archae	87
B0R6B7	archae	87	Q1PCB3	animal	83	A8NPN6	animal	98
B7L830	bacA	86	A0YEN2	bacOther	90	Q2J2E8	bacA	90
Q1V4W4	bacA	85	C1V6J5	archae	73	O49962	plant	90
B7ABU8	bacOther	85	B2FH51	plant	87	C2G2V2	bacOther	87
B7ABU5	bacOther	88	A4SA33	plant	95	Q43701	plant	90
A0QK17	bacOther	87	B1T780	bacOther	91	C2RXB8	bacOther	90
C2IEN7	bacA	85	C1BSB7	animal	83	Q970D9	archae	87
B3YEC9	bacA	86	B6SRC0	plant	85	B3DNV4	bacOther	107
C2N0D2	bacOther	92	A3MGW0	bacOther	90	Q5ENY9	animal	83
A9H2E8	bacA	86	Q312K2	bacOther	85	Q8LMQ7	plant	86
A4AL66	bacB	88	C3MF18	bacA	92	A9LPA2	animal	83
Q41218	plant	92	B4RCJ8	bacA	88	B4TTN0	bacOther	88
B0C7V1	bacOther	89	A1VPN1	bacOther	91	A1TWP0	bacOther	89
B6SZ50	plant	97	A2V217	bacA	86	A8NRR9	fungi	119
A5Z8K7	bacOther	98	C2MKC3	bacOther	91	A2X9T6	plant	84
Q18KD0	archae	73	A2XEH6	plant	92	B3HA30	bacA	86
Q1HAT5	animal	83	O18634	animal	83	B0HTL6	bacA	86
A2QFQ8	fungi	97	B9GYA4	plant	81	B0HTL7	bacA	86
Q1MBV5	bacA	87	P27777	plant	90	C0GNC9	bacOther	89
B8DSE1	bacOther	88	A3CA50	plant	98	Q7PT53	animal	83
B0SS64	bacOther	89	Q6AM30	bacOther	89	Q7PT52	animal	113
C2HSI2	bacA	85	P46729	bacOther	87	B5CKF8	bacOther	88
Q8ZL03	bacA	86	Q11EQ2	bacA	87	A9CPE4	animal	83
B4JME7	animal	125	B7JMI9	bacOther	90	B0JUE5	bacOther	89
A5B2N0	plant	90	Q3Z7X0	bacOther	89	A8GRF0	bacOther	85
A8IXN9	plant	91	Q0UWH5	fungi	139	A5VL40	bacOther	90
A9FA72	bacOther	89	A9ADY4	bacOther	89	B3NCE9	animal	83
Q2NBM0	bacA	87	C1BPD5	animal	84	A9DJ70	bacA	86
A2W504	bacB	92	B6DZ38	animal	83	Q2KD97	bacA	90
A2W503	bacOther	88	A9NX13	plant	92	A7QKW7	plant	90
Q13ER7	bacA	90	Q15PV4	bacOther	90	A1AHM1	bacA	86
B4NBT3	animal	126	Q9ZP24	plant	87	Q890A7	bacOther	90
A2R5S8	fungi	90	Q16S79	animal	83	A7ABY8	bacOther	92
A3IXT1	bacOther	89	C2ZP48	bacOther	92	B3NCE7	animal	92
Q1NI79	bacB	89	A9NS87	plant	90	A9CFS9	bacA	88
P02509	animal	83	B4MAS6	animal	125	B7PWF4	animal	78
B2TGK1	bacOther	89	B3WMM1	bacA	86	B6I3R9	bacA	86
Q7RS01	other	86	B3WMM2	bacA	86	Q19AL9	fungi	131
P02505	animal	83	A3VTU6	bacA	87	B3CLF4	bacOther	89
P02504	animal	83	Q1R4P9	bacA	86	Q2JPN1	bacOther	88
A5FBJ9	bacOther	90	C8CBN4	animal	105	Q7S0S7	fungi	138
P02501	animal	83	A9NMF4	plant	96	B9IN54	plant	72
P02503	animal	83	A7GPA2	bacOther	92	C6TDW8	plant	77
P02502	animal	83	B1FD39	bacOther	91	A5UUL5	bacOther	88
B2UG24	bacOther	91	P0C059	bacA	86	Q6HJB3	bacOther	91
B2UG25	bacOther	88	A2RBG6	fungi	137	B8ZS71	bacOther	87
Q5V1J3	archae	87	Q9SYG1	plant	95	A2Z7R5	plant	83
Q1BXG4	bacOther	88	A5JZ38	other	91	Q9SYU9	plant	90
Q1BXG3	bacOther	91	B8CPJ4	bacA	86	Q9SYU8	plant	90
A6U4B5	bacOther	86	Q9RNV9	bacB	90	C0GCW4	bacOther	89
Q69BI7	plant	90	B4IYY1	animal	118	B2ZES2	plant	90
A3V7F6	bacA	86	B4IYY0	animal	93	Q4FP87	bacA	90
A6Q5H5	bacOther	89	P19037	plant	90	A6M0V3	bacOther	88
Q6EMB9	plant	87	P19036	plant	90	Q8S1F2	plant	74
A9NTL9	plant	91	A0M3R4	bacOther	91	Q0ABW2	bacOther	89
Q63BU3	bacOther	91	B9IQG7	plant	90	C4ILE7	bacOther	88
B5G211	animal	88	Q07UJ0	bacA	90	O74984	fungi	90
A9NMH9	plant	86	B6T339	plant	90	O35878	animal	83
Q0KBR2	bacOther	88	C0SEB0	fungi	100	Q1GBV6	bacOther	90
Q1PE43	plant	85	B5Z5Q4	bacOther	90	Q4KF11	bacA	86
A1UBV4	bacB	88	B9IQG8	plant	90	A5A8U2	plant	87
B1VAS8	bacOther	89	Q40935	plant	91	A1VLW1	bacOther	89
A9NMH6	plant	90	B9BX23	bacOther	88	Q1AMF5	animal	83
A9UEY9	animal	84	Q40936	plant	91	Q5P285	bacOther	90
Q2LU35	bacOther	89	Q6XBS2	plant	90	A4YFP5	archae	68
A7P6D4	plant	94	A7HFL1	bacOther	89	B0ML21	bacOther	92
A9ZKL8	bacB	89	A1DEG0	fungi	92	A5IEW2	bacOther	89
B1I3T2	bacOther	89	A8WY18	animal	82	B0ML22	bacOther	92
A8C8V8	animal	83	B3I5J9	bacA	86	Q2YZ46	bacOther	86
Q96489	plant	90	B3I5J8	bacA	86	A8U8N9	bacB	90
A8KUB3	bacOther	88	Q9ZSX7	plant	94	A7VDD3	bacOther	91

C4QAN1	animal	83	Q07NG0	bacOther	87	Q607M7	bacOther	89
B4PEX9	animal	94	C2YA68	bacOther	90	Q9RNW7	bacOther	90
C2XB98	bacOther	92	A9NY56	plant	90	B5MGV8	bacA	86
C2DJU4	bacA	86	B5P171	bacA	86	B4G197	plant	90
B1ZWT6	bacOther	88	B6FU63	bacOther	92	B5IP54	bacOther	89
B9IZQ3	bacOther	92	A4W4S4	bacA	86	Q9RNW3	bacOther	90
C3N161	archae	75	B8BFT1	plant	85	B2S8F5	bacA	89
A0YUX0	bacOther	89	B0VEU3	bacOther	90	B4RI08	bacOther	86
B9CPV8	bacOther	86	B5RG18	bacA	86	Q81QZ9	bacOther	90
Q2P788	bacOther	89	P02475	animal	83	A9ASQ2	bacB	90
Q2LGT3	archae	79	A5BGB7	plant	90	Q0SYM9	bacA	86
Q0WQZ8	plant	90	Q1MX15	animal	83	C1ZSA2	bacOther	89
A7Q7Q1	plant	91	A0L7B6	bacB	89	B0FFN6	plant	90
B3E7I6	bacOther	89	C1BQM9	animal	92	A8WY04	animal	82
A1KK77	bacB	88	B7CLI1	bacB	91	Q046M7	bacOther	90
A0ACP1	bacB	89	B7CLI0	bacOther	88	B5XB32	animal	87
C3NFM4	archae	87	Q7D412	bacA	85	A8ZNK2	bacOther	89
Q66M65	animal	83	Q9WYK7	bacOther	89	O82012	plant	90
A0Q8Q4	bacB	87	C4KV47	bacOther	88	O82013	plant	90
B9BX22	bacOther	91	A5B3G4	plant	90	O82010	plant	90
B3WBI7	bacOther	90	B2FH45	plant	90	O82011	plant	90
Q88HW9	bacOther	89	A2VRD4	bacOther	88	A5J903	bacB	91
B5C573	bacA	86	A2VRD3	bacOther	91	A5J902	bacOther	88
Q1CD74	bacA	86	A6AUS1	bacA	85	A9P2P1	plant	82
A4WX24	bacA	86	B6KBD2	other	95	A9P235	plant	91
P23927	animal	83	A0YKV7	bacOther	89	B1ZGC2	bacA	84
B9VTS0	animal	83	B5NDS6	bacA	86	A1U9H6	bacB	90
Q68X97	bacOther	85	A6DE36	bacOther	89	B5CPX4	bacOther	91
C3F177	bacOther	90	Q755Q9	fungi	95	A0NLC2	bacOther	90
Q652V8	plant	96	A6GDD9	bacOther	73	A3AGE0	plant	90
B3NCF0	animal	111	B2AC08	fungi	131	B4RI00	bacOther	88
B9VTS8	animal	83	Q6K7E9	plant	99	B4UEC5	bacOther	89
Q9HP50	archae	91	P70919	bacOther	86	C3K047	bacA	104
A8KM01	bacOther	90	C1QD82	bacOther	88	Q2HAM9	fungi	138
A8KM00	bacOther	88	P70917	bacA	85	C6T2N6	plant	90
P23928	animal	83	Q38806	plant	90	Q57FL3	bacA	89
B8IFP3	bacA	84	A6NZ93	bacB	90	B4NML7	animal	128
B1Y9Z3	archae	75	B5WJN8	bacOther	88	C0U9B5	bacB	87
A7URP1	animal	83	B5WJN9	bacOther	89	A3FPF5	plant	94
A7URP0	animal	83	C5MKK0	plant	90	B1I1Q9	bacOther	88
B6JB06	bacA	87	B1L9B3	bacOther	89	B2IEX3	bacOther	90
Q6MHC0	bacOther	78	Q6W1D1	bacOther	64	Q9X3Z5	bacOther	90
A3S7N7	bacA	86	B5WJN7	bacB	92	B7A7M5	bacOther	85
B1H496	bacOther	90	Q1ZUF5	bacA	85	A4R780	fungi	137
A5F489	bacA	85	Q10ZW9	bacOther	89	Q13QP5	bacOther	88
O80432	plant	82	B2FH58	plant	77	B3DXT5	bacOther	91
B9N499	plant	80	B5P172	bacA	86	A7VSV2	bacOther	91
B7IV85	bacOther	91	A4RQ46	fungi	150	B4BD51	bacOther	88
C4HG53	bacA	86	C1TAJ0	bacOther	77	C4ANX3	bacB	91
B7GSP7	bacOther	91	Q10ZW7	bacOther	89	B1YAC1	archae	75
B3N922	animal	91	Q10ZW6	bacOther	89	Q16S81	animal	83
Q9XGS6	plant	90	C0WZY1	bacOther	90	Q16S83	animal	83
B3NCF2	animal	94	B6JCN0	bacA	90	B3LB99	other	96
C2JHV8	bacA	85	B2SNJ9	bacOther	89	Q9HHW1	archae	79
Q8A8R3	bacOther	92	A3NZ89	bacOther	88	Q73ZA7	bacOther	87
B5XBY4	animal	83	B4QN52	animal	90	Q73ZA5	bacB	87
B4LMP4	animal	93	B5YI86	bacOther	89	B4F976	plant	90
B0H718	bacA	86	B4QN50	animal	83	B5K7Q1	bacA	86
B9PKY2	other	95	A4BUZ7	bacB	89	C4I401	bacB	90
Q3T149	animal	83	B3BUL8	bacA	86	A8NLM9	fungi	91
C2A4D7	bacOther	84	A6TFZ0	bacA	86	B3NCE8	animal	84
Q5MCL3	bacOther	81	B0SS63	bacOther	88	A5C6B0	plant	90
B4RI07	bacOther	87	Q47PQ5	bacOther	88	C3H0J4	bacOther	92
B9KZV1	bacOther	89	B5FEV7	bacA	86	A4Z153	bacA	88
A8HNU9	plant	121	Q1R4Q0	bacA	86	B6I3S0	bacA	86
B0H717	bacA	86	B3BUL7	bacA	86	B9SMA2	plant	90
C4HG54	bacA	86	B6A1D5	bacA	85	B6JHC8	bacB	89
Q1J127	bacOther	88	C3R2S0	bacOther	92	B7KTG1	bacA	87
B3ENM6	bacB	89	B3YJK9	bacOther	88	A9P1C1	plant	90
Q89LA9	bacB	89	Q88SP7	bacOther	90	B9HT33	plant	90
A6CA38	bacOther	90	A7FUI4	bacOther	88	Q2TXY8	fungi	90
A1RJ61	bacA	86	C3RP50	bacOther	91	Q3J669	bacA	86
Q3SNF2	bacOther	73	C2R7N9	bacOther	92	A9D1L3	bacA	88
Q3SNF1	bacOther	87	Q4V3R7	animal	116	B4QAX2	animal	93
C1BVY5	animal	84	Q607M9	bacB	91	B1M6Q3	bacA	90
Q1Q0W8	bacB	89	Q607M8	bacOther	88	A9NPZ7	plant	92

Q3SNF5	bacA	85	B6KKL2	other	94	A3WSU3	bacA	85
B4MN44	animal	84	Q9ZDQ3	bacOther	85	A9M7A8	bacA	89
Q3IJ57	bacA	88	B9RL39	plant	80	A6BKD2	bacOther	91
Q0CEJ2	fungi	92	Q6LW17	bacA	86	B4MN48	animal	95
C1NBK1	bacOther	89	Q1PVQ2	bacOther	89	B4MN45	animal	106
C2I247	bacA	85	C3F165	bacOther	91	Q0W782	archae	89
Q92VB0	bacOther	87	A9VVZ8	bacA	87	A3MPF5	bacB	91
Q3IJ58	bacA	88	Q1DDC1	bacOther	89	Q215C3	bacOther	87
Q12MB8	bacA	88	Q6G5E9	bacA	89	B4MN41	animal	83
A6BWH0	bacA	86	Q000T3	animal	83	B1FD38	bacOther	88
C1HSW1	bacA	86	O59514	archae	89	B4MN43	animal	92
Q1QSF8	bacOther	93	Q3A7L5	bacOther	89	P05477	plant	90
Q1QSF9	bacOther	88	B0NTK3	bacOther	92	A6GDE0	bacOther	88
Q5DED1	animal	83	B1CB98	bacB	91	A0LJ28	bacOther	87
C2PVG9	bacOther	93	C3X6V1	bacOther	89	Q6GE45	bacOther	86
B3NCE5	animal	116	Q47QL7	bacOther	87	P05478	plant	90
A8N4X3	fungi	91	B9RQ28	plant	79	B1XQB7	bacOther	89
A3CJL3	plant	82	Q39RC3	bacOther	89	Q8GV39	plant	89
B3AXF8	bacA	86	Q472U3	bacB	90	Q3JTX7	bacOther	90
Q7QIZ9	animal	83	B5EBV7	bacOther	89	A5P7P9	bacA	104
A6AHJ3	bacA	85	P0A5B7	bacB	88	C3EKB4	bacOther	90
B4H1L8	animal	95	C4E0P4	bacB	106	Q16082	animal	83
B9HVH4	plant	77	B0Y732	fungi	139	Q5WTV9	bacOther	89
A9SJI0	plant	90	Q4E663	other	90	Q8GV37	plant	96
C2DJU5	bacA	86	Q68DG0	animal	80	Q8GV36	plant	96
Q88LF1	bacA	87	Q1NNW6	bacOther	89	Q8GV35	plant	96
B2JH61	bacB	90	P0A5B8	bacB	88	Q46E12	archae	72
Q6MA71	bacOther	87	Q9VSX2	animal	84	Q38815	plant	91
B7FXQ8	other	83	B0WER2	animal	83	Q86I25	other	116
Q0ALQ6	bacA	88	B0WER0	animal	83	O53673	bacB	97
A2ST07	archae	92	B0WER1	animal	83	A0KDX1	bacOther	88
Q18E08	archae	90	Q12329	fungi	93	B9AME3	bacOther	92
Q69XV5	plant	82	A4KNR9	bacB	97	O64960	plant	84
B5QLF7	bacOther	90	B9R0U1	bacA	90	Q94KM0	plant	89
A8ACK0	bacA	86	Q46E11	archae	73	B9S391	plant	90
B7NQY7	bacA	86	Q17102	animal	85	B9S392	plant	90
Q9HSS6	archae	73	Q3SGI5	bacOther	90	B9S393	plant	90
A8XSJ6	animal	82	Q6G023	bacA	88	B9S395	plant	90
P80485	bacOther	90	A5YJE5	plant	90	A9NYM3	plant	90
A6GNZ7	bacA	85	C6TKZ3	plant	79	B7ICZ9	bacOther	89
Q47IE4	bacOther	90	A4CCV3	bacA	89	A9S5S2	plant	90
B8M7E8	fungi	158	C2IXH3	bacA	85	C3N952	archae	75
A0B9M4	archae	84	A9MDG8	bacA	88	Q8TG38	fungi	125
A6WNX5	bacA	86	Q2YP08	bacA	89	Q98II6	bacOther	87
B2FH49	plant	85	Q9XET1	plant	90	B4LH14	animal	83
Q11G10	bacB	89	C2FK47	bacOther	88	Q40826	plant	90
B9HPA6	plant	90	C0D4C2	bacOther	90	Q10P60	plant	92
B9HYD1	plant	91	B4L109	animal	84	C0GJK2	bacOther	89
A4EXD4	bacA	86	Q8EET9	bacA	86	Q5SHV9	bacOther	89
Q0VNM3	bacA	87	C3H0K5	bacOther	90	A9NN36	plant	76
A8XV10	animal	82	A2PUK5	bacA	85	A3NZ90	bacB	91
Q2W556	bacA	86	Q00649	animal	83	B6TTC8	plant	90
C8CBN5	animal	91	Q9PBB0	bacOther	89	C4GZC1	bacA	86
Q0ZLZ4	animal	83	Q1QM22	bacB	89	C4GZC0	bacA	86
C1Q5Y1	bacB	88	C0WBL1	bacOther	91	Q98II5	bacA	85
A8XV11	animal	82	B7KV94	bacA	84	A9PE56	plant	81
Q604K7	bacB	89	B5N0S9	bacA	86	B8D8E3	bacOther	88
A8EEY2	bacOther	90	A3SQG5	bacA	86	Q2JDT4	bacOther	90
A8EEY3	bacOther	88	A7IJ68	bacA	88	Q1D4I0	bacOther	89
A8XV12	animal	82	A0MWF1	plant	90	Q0BUB1	bacOther	86
C1V6Z4	archae	89	C4CHL2	bacOther	92	B2H0I1	bacB	91
C3KMX0	bacOther	89	Q7YWK0	animal	82	A5I2N2	bacOther	88
C3KMX1	bacOther	88	Q3SIX7	bacOther	89	B7K4Y3	bacOther	89
C3KMX2	bacOther	89	A2XEX0	plant	90	B2H0I2	bacOther	88
C1V6Z3	archae	93	A2XEX1	plant	90	A4EEB5	bacA	86
C1YN50	bacOther	87	A9R4H2	bacA	86	Q4LBZ8	bacOther	92
Q6CD64	fungi	93	C3N3M5	archae	87	C4INX5	bacA	89
B5G208	animal	88	A8MB44	archae	75	Q8THE4	archae	72
A0K688	bacOther	91	A9RGK8	plant	90	A9T894	plant	92
Q0ZLZ3	animal	100	Q8UUZ6	animal	83	B5XCL5	animal	87
A0K687	bacOther	88	B1I4G5	bacOther	90	Q3A9G8	bacB	90
C2F915	bacOther	90	B9CG20	bacOther	85	A4IJW6	bacOther	88
A8EAQ6	bacB	90	B7NF04	bacA	86	C3RDB9	bacOther	93
Q3JIL4	bacB	90	B7NF03	bacA	86	B5DFY5	animal	83
Q5ENZ0	animal	83	Q1QRS9	bacA	92	Q3B693	bacB	89
A1UXF5	bacB	90	A1T4V8	bacB	88	C4B4U5	animal	83

Q10NK9	plant	90	C3FJQ8	bacOther	91	C0QVM2	bacOther	99
Q5FMH4	bacOther	90	A7JF86	bacB	87	Q9HP75	archae	87
A3D568	bacA	86	Q2H8B0	fungi	166	C4J494	plant	90
Q10NK3	plant	90	A1CLF4	fungi	126	B3AGV5	bacA	86
Q10NK2	plant	90	Q5B0Z9	fungi	148	O26947	archae	89
Q10NK1	plant	90	A4YGA0	archae	87	Q7PCM8	animal	90
B4GHU4	animal	128	A6WVA1	bacA	89	C1BEB6	bacOther	87
Q2IDH5	bacOther	89	B1KQJ8	bacA	86	C4CVV2	bacOther	93
B4S0S7	bacA	91	B9SE69	plant	95	B3PU08	bacA	84
C0G910	bacA	88	B5ED45	bacOther	88	B3PU09	bacOther	87
C3LP91	bacA	85	C6T116	plant	92	C2EQJ9	bacOther	90
Q0HJE5	bacA	86	Q1LU49	bacOther	92	B6K8V8	other	95
B9GPN0	plant	82	Q127W4	bacOther	74	O34321	bacOther	86
P70918	bacA	85	C3QYQ3	bacOther	92	A7R0M1	plant	80
B3EGS0	bacOther	89	B6QBD8	fungi	101	P68282	animal	83
B4IYY2	animal	84	B7FMD9	plant	90	Q8ZVI0	archae	88
B9SSG1	plant	83	A8RAG0	bacB	91	B4SD99	bacB	89
A7QY87	plant	82	A4FYB5	archae	90	Q6A9S3	bacOther	88
A7QY88	plant	82	Q749R4	bacOther	89	Q30XK2	bacOther	89
A7MPY9	bacA	86	C1DSQ9	bacA	91	Q6CJG1	fungi	95
B8EMT0	bacA	90	A4MH11	bacOther	90	B0XZX3	fungi	124
Q9ZS24	plant	90	A4MH12	bacOther	88	A3PT33	bacB	90
C4QAM8	animal	73	A8F137	bacOther	85	Q02PT9	bacA	87
C4QAM9	animal	83	Q2UMF7	fungi	143	A6USD0	archae	90
B2D2G4	plant	88	Q1GW59	bacOther	88	B7UMF4	bacA	86
B8IBB5	bacB	87	Q1GW58	bacOther	89	B3Q3L7	bacOther	85
C4QAM4	animal	83	A8ACJ9	bacA	86	P68284	animal	83
Q645R1	animal	83	B0ML08	bacOther	92	A4SH54	bacA	85
Q1N5J3	bacOther	89	C3LJL7	bacOther	91	Q2NAA8	bacA	104
B7BA63	bacOther	92	C1M216	animal	73	Q0FLW4	bacA	86
Q8KEP2	bacOther	89	C2K1C1	bacOther	90	A3Z2E5	bacOther	89
O52192	bacOther	90	C2GG58	bacOther	88	B0CJ23	bacA	89
O52190	bacOther	90	Q2BML8	bacA	86	A9NSY4	plant	96
B4TMX6	bacA	86	Q13ZX0	bacOther	91	Q21062	animal	82
B7C7Z6	bacOther	87	Q28LZ5	bacOther	86	A3JR95	bacA	86
C2FQM0	bacOther	90	Q3YWC6	bacA	86	B9SWM7	plant	90
B4Q8N8	animal	91	Q3YWC5	bacA	86	Q07TU1	bacA	87
C4M4U3	other	87	Q00SF7	plant	97	A5JV83	animal	86
Q82T55	bacOther	89	Q5S1U1	animal	83	A8UL15	bacOther	90
A9P1P3	plant	91	B8JCV4	bacOther	89	A6C3F8	bacOther	93
C1BNN7	animal	92	P34328	animal	83	B4QMS5	animal	116
A0LKU5	bacOther	88	C0Y6K1	bacOther	88	B8DSE0	bacB	85
Q5AUY8	fungi	127	Q5H9M9	animal	94	Q96458	plant	90
B4I777	animal	124	C1ASG3	bacOther	87	C1BQF4	animal	92
A1U646	bacOther	89	Q9XIE3	plant	90	C1ME19	bacA	86
Q76NU5	other	148	A9NQG6	plant	82	B3YT47	bacOther	91
A6T455	bacA	86	B5ZE07	bacOther	87	Q9K4W0	bacOther	89
A9KFM9	bacB	89	B7VGI8	bacA	85	Q66KY8	animal	83
Q2NKA9	bacOther	89	Q3J7L3	bacOther	89	B5MZS3	bacOther	88
C4ESC1	bacOther	89	Q3J7L2	bacOther	89	A7JEZ3	bacB	87
A2WRY7	plant	74	B1M1T9	bacA	85	Q48KA2	bacA	87
A2WRY5	plant	86	B0Q3H2	bacOther	90	A5GPG4	bacOther	96
C3R7G3	bacOther	96	B4TFM8	bacOther	88	Q8Z9V6	bacA	86
Q1WDN9	animal	86	Q0GC56	animal	83	B6Q7Z4	fungi	148
Q1WDN8	animal	86	A4BTG1	bacOther	89	B9PGE9	other	95
A1R411	bacOther	88	Q1H253	bacOther	66	Q4UKR8	bacOther	89
A5FDF8	bacOther	90	Q9A2G9	bacOther	90	Q4UKR7	bacOther	85
A5C6A9	plant	90	B9GXR5	plant	94	B2G8H3	bacOther	90
Q15RT9	bacA	87	B5RG17	bacA	86	Q16JG8	animal	83
Q2Y862	bacOther	89	Q06EY3	animal	83	Q9ZSR6	plant	92
C1A981	bacOther	89	B1ZJ37	bacA	86	Q94GC5	plant	79
Q2LYH4	bacOther	89	B5KBA9	bacA	90	B7KQ11	bacA	86
C1ETC8	bacOther	90	B2JR94	bacOther	89	C6T1D0	plant	90
Q0KA57	bacOther	90	C1C0M7	animal	83	A2TUE0	bacOther	90
C0G3T6	bacA	89	B2JR93	bacOther	88	Q5R1P7	animal	83
A0PN37	bacOther	93	A9K3M8	bacOther	90	A9NKY0	plant	86
B5V071	bacOther	90	Q3A7M7	bacOther	88	Q0RIJ5	bacOther	87
P0C058	bacA	86	C2T0Z5	bacOther	92	C7BVR3	animal	82
Q0FDC5	bacA	87	B5Q031	bacOther	88	Q9SXZ0	plant	89
A7P106	plant	91	B1HHR7	bacOther	90	B6TQD6	plant	96
Q5XJD0	animal	80	Q2A147	bacOther	87	A5IDZ0	bacA	87
A2SFL9	bacOther	90	A4IQ30	bacOther	90	B8CZF2	bacOther	88
A9MWJ6	bacA	86	Q012E4	plant	99	P24632	plant	90
A9MWJ7	bacA	86	B4QN53	animal	111	P24631	plant	89
Q2FVN2	bacOther	86	A7QRP2	plant	90	A8DR35	plant	85
B4RED2	bacB	89	A7QRP1	plant	90	B0ET55	other	84

B4A4X1	bacA	86
B4A4X0	bacA	86
B9HHJ3	plant	90
A4S8A2	plant	99
Q1AMF6	animal	83
Q1AMF7	animal	83
B1Y9D3	archae	88
Q6CQF7	fungi	93
A8L1Y9	bacOther	90
Q6SJQ8	animal	142
B5C574	bacA	86
Q30T88	bacOther	89
B7RMY8	bacA	86
B9QE19	other	94
C2TWX1	bacOther	92
B4KHI6	animal	91
Q30T87	bacOther	90
B8DIS6	bacOther	85
B9H7S6	plant	95
B9R659	bacA	86
C0BAE3	bacOther	92
C0BAE2	bacB	91
Q5E4T5	bacA	86
Q03U39	bacB	90
C3QDE3	bacOther	92
C3MU73	archae	75
Q5VRY1	plant	94
B9JZK1	bacA	91
C4WSK0	animal	83
A6L6R8	bacOther	93
B6U6V5	plant	91
Q5DI11	animal	83
Q2IHQ7	bacOther	89
C2UDD0	bacOther	90
P46254	plant	84
C2WLX3	bacOther	92
A8P0X0	animal	83
B5QX88	bacOther	88
A2SHG1	bacOther	91
P42930	animal	83
P42931	animal	87
B5UU71	bacOther	90
Q98IT5	bacOther	87
B2YI77	archae	87
A5B3K5	plant	90
A5B3K6	plant	80
A4KIH4	bacB	88
C1BU38	animal	84
Q5TWA3	animal	116
Q3EM33	bacOther	91
C4M7B0	other	87
B0A7M1	bacOther	91
B0A7M0	bacOther	91
B3VMZ8	other	97
B3VMZ9	other	97
A9P0Z1	plant	82
A9L3Q0	bacA	86
Q9FY38	plant	90
A1ZJ14	bacOther	96
C3NTM2	bacA	85
Q9SWE4	plant	90
C1SRV5	bacOther	88
C1SRV4	bacB	85
B5D2J0	bacOther	92
Q2JUK4	bacOther	89
Q9GT43	animal	81
Q5FW72	animal	80
A8TSP0	bacA	85
A9NLK7	plant	90
B2J4L6	bacOther	89
A9P1S2	plant	90
Q9P9K5	archae	89
A3VC52	bacA	86
A8UZP7	bacOther	86
B7K5D6	bacOther	89
B8BFT0	plant	85
B6T2J9	plant	90
A3MIL4	bacOther	90
Q1CD75	bacA	86
B4UKF4	bacOther	89
B1L3F9	archae	73
B6IW95	bacA	85
Q6Z6L5	plant	97
A3MIL3	bacOther	88
Q9PUR2	animal	83
B2W1D8	fungi	200
C1ETB6	bacOther	91
Q974V6	archae	73
Q8MMN1	other	154
A1WZ40	bacOther	89
A7T2X8	animal	97
B3KQL3	animal	80
A9S018	plant	86
A3CX48	archae	90
B8GSY7	bacOther	89
C4KF22	archae	87
Q57V53	other	90
A8P845	animal	83
B4TA61	bacA	86
B4TA60	bacA	86
A7ZTP0	bacA	86
B3ZA61	bacOther	90
C0QIP5	bacOther	89
B7HQ44	bacOther	92
B4MN46	animal	111
Q3ADV9	bacOther	90
Q58GG5	bacOther	89
P05812	animal	110
C0K035	bacOther	93
P05811	animal	83
B3HA31	bacA	86
B7N2D2	bacA	86
B8LM67	plant	91
A8WY05	animal	82
Q08V45	bacOther	89
Q06EY2	animal	83
C0VRF8	bacOther	88
A9NDP5	bacB	89
Q9SHJ1	plant	86
A5A8T6	plant	89
A6UD15	bacA	87
B8M0J1	fungi	101
B1N202	bacOther	88
B3K3L8	bacB	90
C2YR86	bacOther	92
Q052T2	bacOther	87
Q1GW60	bacOther	89
A7A326	bacOther	91
A5Z9G6	bacOther	91
Q1GW65	bacA	87
Q1GW66	bacOther	87
A5A8T5	plant	89
A7ZTP1	bacA	86
Q9BHC0	other	95
B0UG17	bacOther	91
C3NME5	archae	75
A9NLE2	plant	82
O81822	plant	91
A5CST5	bacOther	88
Q5LMS8	bacA	86
Q13ZW0	bacOther	95
C0YQ53	bacOther	90
